# A comparison of PTI defense profiles induced in *Solanum tuberosum* by PAMP and non-PAMP elicitors shows distinct, elicitor-specific responses

**DOI:** 10.1371/journal.pone.0236633

**Published:** 2020-08-12

**Authors:** Rafaela Lopes Martin, Pauline Le Boulch, Pauline Clin, Adrián Schwarzenberg, Jean-Claude Yvin, Didier Andrivon, Eric Nguema-Ona, Florence Val

**Affiliations:** 1 AGROCAMPUS-OUEST, UMR IGEPP 1349-Institut de Génétique, Environnement et Protection des Plantes, Rennes, France; 2 Centre Mondial de l’Innovation Roullier, Laboratoire de Nutrition Végétale, Pôle Stress Biotiques, Saint Malo, France; 3 INRAE, UMR IGEPP 1349-Institut de Génétique, Environnement et Protection des Plantes, Le Rheu, France; University of Nebraska-Lincoln, UNITED STATES

## Abstract

The induction of general plant defense responses following the perception of external elicitors is now regarded as the first level of the plant immune response. Depending on the involvement or not of these molecules in pathogenicity, this induction of defense is called either Pathogen-Associated Molecular Pattern (PAMP) Triggered Immunity or Pattern Triggered Immunity—both abbreviated to PTI. Because PTI is assumed to be a widespread and stable form of resistance to infection, understanding the mechanisms driving it becomes a major goal for the sustainable management of plant-pathogen interactions. However, the induction of PTI is complex. Our hypotheses are that (i) the recognition by the plant of PAMPs *vs* non-PAMP elicitors leads to specific defense profiles and (ii) the responses specifically induced by PAMPs target critical life history traits of the pathogen that produced them. We thus analyzed, using a metabolomic approach coupled with transcriptomic and hormonal analyses, the defense profiles induced in potato foliage treated with either a Concentrated Culture Filtrate (CCF) from *Phytophthora infestans* or two non-PAMP preparations, β-aminobutyric acid (BABA) and an *Ulva* spp. Extract, used separately. Each elicitor induced specific defense profiles. CCF up-regulated sesquiterpenes but down-regulated sterols and phenols, notably α-chaconine, caffeoyl quinic acid and rutin, which decreased spore production of *P*. *infestans in vitro*. CCF thus induces both defense and counter-defense responses. By contrast, the *Ulva* extract triggered the synthesis of a large-spectrum of antimicrobial compounds through the phenylpropanoid/flavonoid pathways, while BABA targeted the primary metabolism. Hence, PTI can be regarded as a heterogeneous set of general and pathogen-specific responses triggered by the molecular signatures of each elicitor, rather than as a uniform, non-specific and broad-spectrum set of general defense reactions.

## Introduction

Plants have developed a complex immune system to protect themselves against pathogens and other biotic or abiotic stresses. The interactions between plants and pathogens are now classically described as a co-evolutionary arms race designated as the zig-zag model [1]. The first level in this immune system is based on the recognition by the plant of exogenous molecules or molecular patterns, which the plant uses as signatures of local disturbances of its environment. Among these molecules are pathogen/microbe-associated molecular patterns (PAMPs/MAMPs)—*i*.*e*. molecules produced by the pathogens or microorganisms and key to its survival and fitness [2], but also other exogenous defense elicitors not related to pathogenicity. The zig-zag model assumed that both types of elicitors (PAMPs or non-PAMPs) would trigger the activation in the plant of general defense mechanisms [3], a process designated as either PAMP Triggered Immunity or Pattern Triggered immunity (PTI) according to the origin, nature and function of these molecules [4]. PTI is therefore commonly thought to be responsible for non-host resistance (incompatible interactions), and for quantitative resistance (compatible interactions) [3]. In addition, over the course of evolution, hosts have sometimes also developed the ability to recognize specific avirulence factors. This recognition triggers a second level of the immune system, effector-triggered immunity (ETI), which leads to the induction of more intense defense responses than PTI. ETI is generally considered to drive qualitative, gene-for-gene resistance [5], which is generally less durable than the quantitative forms of resistance associated with PTI [6].

Because it is assumed to be a widespread, broad-spectrum and stable form of plant resistance to stress, including pathogens, PTI is now receiving renewed attention, and understanding the mechanisms behind it becomes a major goal to sustainably engineer and manage plant health [7]. Plants might perceive PAMPs/MAMPs alone or as complex mixtures containing several active elicitors. This is the case for flagellin 22 (flg22) [8], lipopolysaccharides and peptidoglycans [9] from bacteria, or chitin [10] from fungi, but also of Concentrated Culture Filtrates (CCF) of the potato late blight pathogen, the oomycete *Phytophthora infestans* containing several PAMPs [11]. The recognition/perception by the plant of such elicitors, alone or as mixtures, can happen in different ways. Some receptors specific to particular PAMPs have been identified. For instance, Zipfel *et al*. [12] showed that the resistance in *Arabidopsis* to *Pseudomonas syringae* pv. *tomato* depends on the perception of flg22 by the Flagellin-Sensing receptor FLS2. Furthermore, intracellular signaling also depends on the co-receptor Brassinosteroid insensitive-associated kinase 1 [13]. These studies complemented earlier results by Gómez-Gómez *et al*. [14], demonstrating the nature and sequence of defense‐related responses triggered by flg22: ROS burst minutes after the treatment, followed by callose deposition and PR-1 and PR-5 expression after 24h.

Besides PAMPs, the plant immune system can be activated by other exogenous elicitors. Such exogenous elicitors include phytohormones or their analogues, such as salicylic acid analogue acibenzolar-S-methyl in tobacco [15]. Exogenous elicitors can also be plant cell wall degradation products, released when tissue is injured mechanically or upon biological stress (Damage Associated Molecular Patterns–DAMPs) [16], or oligosaccharides/polysaccharides directly extracted from plants or algae such as ulvan [17] or laminarin [18]. Some exogenous elicitors prepare the immune system by accumulating and storing molecules that can serve the defense against pathogens [19]. For instance, in potato, β-aminobutyric acid (BABA) can prime the synthesis of defense molecules, which are then directed to the pathogen after the infection [20]. These molecules, highly diverse in nature, can activate metabolic pathways involved either in defense pathways common to biotic or abiotic stress [21] or in defense responses specific of pathogen infections [22–24]. PTI can thus be understood and defined as “defense induced upon initial exposure of plants to molecular patterns, produced by pathogens (PAMPs), other microbes (MAMPs) or resulting from cell damage (DAMPs) after recognition of these molecular patterns by the plant”. This definition was used in this paper.

While most studies on PTI were carried out on the model plant *Arabidopsis thaliana*, limited reports exist on arable crop plants such as potato. These previous studies show that potato can perceive different defense-inducing molecules, including exogenous elicitors such as BABA [25, 26] and the PAMPs present in CCF of *P*. *infestans* [27]. The treatment of potato tubers [28, 29] or potato leaves [30, 31] with CCF induce the phenylpropanoid pathway, particularly *PAL* (phenylalanine ammonia-lyase) and *PR* genes. CCF also induced chlorogenic acid accumulation in potato tubers [28, 29] and the expression patterns of defense and the SA pathway [30] according to genotypes, and when applied prior to inoculation with the pathogen, promotes the reduction of late blight symptoms depending on *P*. *infestans* isolates [31].

Our working hypotheses are thus that 1) the recognition of PAMP *vs* non-PAMP elicitors by the plant can lead to differential defense profiles, characteristic of the molecular signature of the elicitor involved, and 2) the responses specifically induced by PAMPs target critical life history traits of the pathogen that produced them. One strategy to test them is to compare the defense patterns induced by PAMPs and non-PAMPs elicitors in the same plant material. We thus choose for this work one plant species (the cultivated potato *Solanum tuberosum*) and three elicitors preparations: i) CCF, containing at least 3 α-elicitins (INF1, INF4 and INF5) and a galactan-based complex polysaccharide [11], which mimics the perception of *P*. *infestans* [31]; (ii) BABA, a synthetic, non-protein amino acid, and (iii) an *Ulva* extract composed mainly of ulvan, a complex polysaccharide made of rhamnose, glucuronic acid, and iduronic acid, sometimes decorated with sulphur [17]. Many authors have shown the efficacy of BABA against *P*. *infestans* on potato genotypes under laboratory [25, 26] and field conditions [32, 33], with a significant reduction in symptoms. In addition, BABA has a potentiation effect to trigger the defense pathways by accumulation of transcription factors, of signaling proteins and changes on the metabolome [20]. For its part, the *Ulva* extract has been shown to induce defense reactions and reduce disease severity in various pathosystems, with best results observed against the powdery mildew agents *Erysiphe polygoni*, *E*. *necator* and *Sphareotheca fuliginea*, in common bean, grapevine, cucumber, respectively [34] and in tomato [35]. Defense responses were analyzed using a metabolomic approach supplemented by a transcriptomic analysis and phytohormone quantification, and the antimicrobial activity of key metabolites produced was measured *in vitro* against *P*. *infestans*.

## Materials and methods

### Preparation of elicitors and plant material

CCF was produced from *P*. *infestans* strain 14-P29-03R, maintained in our in-house collection of fungi on pea agar, according to the protocol developed by Desender *et al* [27] and adapted by Thomas *et al*. [31]. Briefly, *P*. *infestans* mycelium was grown in sterile pea broth for three weeks at 15°C in the dark and under agitation. The culture broth was filtered on sterile non-woven compresses to separate the mycelium from the culture broth, freeze-dried for 72 hours and stored at -20°C until used. The *Ulva* extract was produced from *Ulva* spp, harvested in the English Channel in the region of Brittany and autoclaved for 2h at 90°C, as described by Jaulneau *et al*. [17]. BABA (97%) was purchased directly from Sigma-Aldrich France (CAS 541-48-0).

Tubers from potato (*Solanum tuberosum* L.) genotype Désirée, moderately resistant to *P*. *infestans* [36], were produced in 2016 by BrACySol (UMR IGEPP, Ploudaniel-France). The tubers were germinated 2 weeks before planting, and grown as described by Thomas *et al*. [31]. Briefly, acclimated tubers were grown for 4 weeks in 2L plastic pots in commercial culture substrate (Falienor, Terreau de France NFU44-551). This substrate contains 60% of blond peat, 40% of sand, 40 kg.m^-3^ of clay, 700 g.m^-3^ pg Mix 14-16-18 of NPK. The plants were fertilised with an NPK solution during the second and third weeks and were maintained in a greenhouse at 20±5°C with a photoperiod of 16:8h for a day-night cycle.

### Elicitor application and sample collection

CCF and *Ulva* extract were used at 8 mg/ml in all elicitation treatments. BABA was used at 0.5 mM. All elicitors were diluted in water supplemented with 0.1% “Tween20” for better tissue absorption. In all experiments, 0.1% “Tween20” in water was used as the control.

Each elicitor/control solution was sprayed separately until runoff on 6 potato plants. Forty–eight hours after treatment, 24 leaflets were collected from the middle part of each plant. The 48 hour post treatment timepoint was chosen based on earlier data showing that it the is most suitable to detect both defense gene expression, enzymatic activities and PR expression [30, 37]. These leaflets were pooled and immediately deep-frozen in liquid nitrogen, resulting in six replicates per treatment. Frozen samples were lyophilized and ground in a FastPrep®-24 Classic Instrument for 3 min at 4.0 m/s (MP Biomedicals, France). They were stored at -80°C until analysis. Three independent trials were performed, for a total of 18 samples (3x6).

### Metabolite extraction

Metabolites were extracted separately from each of the 6 samples from two of the three independent trials. The extraction was performed at 4°C, using 10 mg of lyophilized material per sample and using an ice-cold extraction solvent (70% methanol, 29% water and 1% formic acid; all solvents used were LC-MS grade), known to extract mainly the polar and semi-polar secondary metabolites [38]. The extraction began by stirring lyophilized tissue in 1 ml of extraction solvent for 1 min at 30 Hertz, using two 3 mm stainless steel balls to ensure optimal homogenization of plant tissues (Mixer Mill MM400, Retsch, Germany). Samples were vortexed for 30 min and centrifuged for 20 min at 18000 g at 4°C (Eppendorf Centrifuge 5427 R, Hamburg, Germany). Supernatants were collected and pellets were again stirred in 500 μl of ice-cold extraction solvent, vortexed for 5 min and centrifuged for 10 min in the same conditions. The supernatants from this second extraction were collected and added to those of the first extraction, and 300 μl were transferred into LC-MS (Liquid Chromatography-Mass Spectrometer) vials.

### UPLC-QTOF analysis

Data acquisition was performed using an Ultra-High-performance Liquid Chromatography-Quadrupole Time-of-Flight Mass Spectrometer—Xevo G2-S QToF (UPLC-QTOF-MS^e^ from Waters^®^ –Milford/Massachusetts/USA) equipped with an electrospray ion source (ESI). Separation was performed using a Kinetex EVO C18 column (150 x 2.1 mm– 2.6 μm, 100Å, Phenomenex, USA). The metabolites were separated with a gradient elution consisting of a mobile phase (A) Water + 0.1% formic acid and (B) Acetonitrile + 0.1% formic acid, according to a linear gradient of 99% A to 100% B at 0–14 min, 14–18 min at 100% B, 18–20 min at 99% A. The flow rate was 0.6 ml/min, and the column temperature was maintained at 35°C. The samples were stored at 10°C before analysis and they were injected at a volume of 7μl. The acquisition was performed using both ESI negative and positive (ESI- and ESI+) ionization modes. The source voltage was set to 2.5 kV for ESI- ionization, 3.0 kV for the ESI+ and the cone voltage to 30 V for both ionizations. The temperature of the source was stabilized at 120°C with a cone gas flow rate of 20/h. The desolvation temperature was 550°C with desolvation gas flow rates of 900/h. Lock spray, Leucine Enkephalin (Waters, Manchester, UK), was injected at 10 μl/min for real-time data calibration at 1 ng/μl. Before sample injection, the column was cleaned with three consecutive injections of the solvent extraction solution. For each treatment, samples were analyzed individually. The injection order was randomized to avoid the effects of instrumental drift. To ensure analytical robustness and correction of potential instrumental drift, a quality control solution (QC) was prepared with an equal volume mixture of all samples and introduced in the sequence after each fifth sample analyzed. Acquisition mass range was 50–900 Da in a time range of 0–15 min and a scan rate of 0.1 sec. MS data were recorded in profile mode and the resolution was set at 30000 full width half maximum (FWHM). MS^e^ data were recorded using an energy ramp from 30 to 45 V. Final recorders used to the followed analysis were the first spectrometer or MS1.

To avoid any misinterpretation of the results due to the contamination of the extracts with residual, elicitor-associated compounds, elicitors were also treated and analyzed in the same manner the leaflets samples (S1 Fig in S1 File).

### Data acquisition and metabolite identification

All metabolomic data were analyzed using Progenesis QI software (Nonlinear Dynamics, Waters®). The first phase of data analysis was an alignment using one QC run chosen for its similarity with the samples, followed by a peak picking and then normalization to build the data matrix. These processes were performed independently in both ESI- and ESI+ ion mode. Statistical analysis was performed on Progenesis QI by one-way analysis of variance (ANOVA) ≤ 0.05 and fold change analysis (FC) ≥ 2, with a false discovery rate correction (q-value) < 0.05 for each sample metabolite. Following these three pre-treatment steps, data was analyzed using the Principal Component Analysis (PCA), a descriptive non-supervised statistical method in order to highlight the major discriminating variance present in the samples. PCA was used to visualize data on the effects of treatments.

Putative identification of metabolites was assisted by an in-house database containing 2981 plant metabolites, compiled from public references libraries (KEGG, PubChem and CHEBI) and Solanaceae references [37, 39]. Discriminant metabolites were validated by chemical formulae with a mass error (ppm) of ±5, isotope similarities and MS^e^ profiles obtained when the fragmentation profile data were available. The putative identification was classed “Level 2”—Putatively annotated compounds—according to the COSMOS Project (Coordination of Standards in Metabolomics - http://cosmos-fp7.eu/msi.html). Negative and positive ionization data were analyzed together after the putative identification. Common metabolites between ESI- and ESI+ needed to be validated under the following criteria: (i) similar retention time and (ii) same induction of elicitors. The new data matrix was visualized using a heatmap analysis based on correlation-based distance using Pearson correlation coefficients and Ward.D2 linkages with the R software version 3.3.3 with the “gplot” package.

The normalized abundance for individual metabolites was represented as histograms. To test whether normalized abundance of selected metabolites differed among the four treatments, ANOVA for Linear Model Fits were performed. Tukey HSD tests were also carried out to reveal which sample groups were different from each other. These analyses were conducted using R software version 3.3.3 with the “FactoMineR”, “ade4” and “Tukey.HSD” packages.

### Targeted metabolite analysis and *in vitro* tests against *Phytophthora infestans*

Some targeted metabolites, classed “Level 1”, from different defense pathways were analyzed in ESI-also using UPLC-QTOF-MS^e^: caffeoyl quinic acid (Sigma-Aldrich France—CAS: 905-99-7) a phenylpropanoid, rutin (Sigma-Aldrich France-CAS: 207671-50-9) a flavonoid and α-chaconine (Carbosynth—Batch n°OC293251601) a glycoalkaloid. The standard curves were drawn from 800 ppb to 50 ppm and metabolites were quantified with the Masslynx sofware (Waters®).

The direct effect, on *P*. *infestans*, of the targeted metabolites was evaluated according to the adapted method from Kröner *et al*. [29]. The standard metabolites were diluted in distilled water to the maximum concentration detected in lyophilized leaflets: 26 μg/ml for α-chaconine, 24 μg/ml for caffeoyl quinic acid and 4 μg/ml for rutin. 500μl of each metabolite solutions or 500 μl of water (negative control) have been spread on the surface of pea agar in a Petri dish. Five replicates were performed per metabolite. The dishes were dried in a laminar flow hood. A 1 cm^2^ explant of mycelium from *P*. *infestans* was deposited in the center of each Petri dish and the dishes were placed in the dark at 15°C. Every day, the mycelium growth zone was delineated with a marker. After 8 days, the Petri dishes were photographed and the mycelium growth was evaluated using the ImageJ software.

The non-destructive measurements of mycelium growth made possible to maintain the Petri dishes in the dark at 15°C to promote the sporulation of *P*. *infestans*. After 3 weeks of culture, the sporangia from each dish were collected in 500 μl of water and then counted with a Malassez cell; from each spore suspension, two independent counts were made.

### Extraction method for transcript analysis

For each biological treatment (elicitor/control) and trial, a pooled sample was obtained by mixing aliquots from the six initial samples. From these lyophilized samples, two 15 mg aliquots were taken for RNA extraction. The details for RNA isolation, RNA integrity checking and cDNA synthesis were as described by Thomas *et al*. [31]. Briefly, samples were extracted with the “SV Total Isolation System” from Promega^®^. RNA concentration was verified in a Nanodrop 2000 (Thermo Scientific^®^), and RNA quality was determined by electrophoresis on 1% agarose gels. The “iScript cDNA Synthesis” from Biorad^®^ was used to generate complementary DNA (cDNA) from RNA samples by RT-PCR with following conditions: (i) 5 min at 25°C; (ii) 30 min at 42°C; (iii) 5 min at 85°C. Then the cDNA quality was verified by electrophoresis on 1.5% agarose gel with the amplification of Pathogenesis-Related proteins 2 (*PR2*) gene.

### Primer designs

The cDNA samples were used to study the transcript accumulation by qRT-PCR for 17 genes selected to represent different steps along the main defense mechanisms: PR protein, phenylpropanoid, flavonoid, alkaloid/terpenoid, Jasmonic Acid (JA), Salicylic Acid (SA) pathways, as well as one housekeeping gene (Glyceraldehyde-3-phosphate dehydrogenase, GAPDH) for normalization purposes (Table 1). These genes are part of the qPFD chip, patented by INRA (WO/2011/161388). Primers were designed using Primer Blast (https://www.ncbi.nlm.nih.gov/tools/primer-blast/) on the potato genome. The sequences of each gene were obtained from NCBI's GenBank and aligned *via* Primer Blast. The selected primers had to meet the two criteria described by Saubeau *et al*. [30]: (i) hybridization temperatures should be between 58 and 62°C; and (ii) the self-complementary index of the two primers in 5' or 3' should not exceed 5 to avoid a non-specific hybridization between the two primers. The effectiveness of primer pairs was assessed with a cDNA dilution range on the basis of two criteria: (i) one peak on melting peaks curves and (ii) qRT-PCR efficiencies from calibration curves (E = 10^[–1/slope]^) between 85% and 120%. The cycling PCR conditions were 5 min at 95°C; 50 cycles of 5s at 95°C, 30 s at 60°C and 30 s at 72°C., with melting curves analysis obtained after 30s at 95°C and 30s at 60°C, as adapted from Saubeau *et al*. [30], in a 384-well Lightcycler480 (Roche). Moreover, the presence and abundance of PCR products was revealed by electrophoresis at 120V on 2% agarose gel with 5x GoTaq buffer.

**Table 1:** Gene’s candidates and their primers sequencing for transcript analysis after the elicitors treatment on potato plants.

Correspondence of metabolic pathway	Gene name	NCBI's GenBank number	Gene function
PR proteins	***PR1 °***	AJ250136	Pathogenesis-related protein 1
	***PR2 °***	U01901	Pathogenesis-related protein 2 (β-glucanase)
	***PR4 °***	AM908515.1	Pathogenesis-related protein 4 (hevein-like)
	***PR5a***	XM_006364057.2	Pathogenesis-related protein 5 (thaumatin like)
Phenylpropanoid pathway	***PAL***	XM_006367472	Phenylalanine ammonia-lyase 1
	***C4H °***	XM_006350825	Trans-cinnamate 4-monooxygenase
	***4CL °***	M62755	4-coumarate-CoA ligase
	***C3H***	BQ513122	P-coumarate 3-hydroxylase
Flavonoid pathway	***CHS2***	NM_001288367.1	Chalcone synthase
Alkaloid and Terpenoid pathways	***HMGR2***	AB041031	Hydroxymethyl glutarate-CoA reductase 2
	***SS1***	JF202610	Squalene synthase 1
	***SGT1°***	NM_001318679.1	Scopoletin glucosyltransferase-like
	***SGT2***	JN695006	UDP-glucose:solanidine glucosyltransferase
	***SGT3 °***	HM188447	rhamnose:beta-solanine/beta-chaconine rhamnosyltransferase
JA pathway	***OPR3***	XM_006358244.1	12-oxophytodienoate reductase 3
	***MeJA***	AY615276	Methyl jasmonate esterase
Transcription factor	***WRKY1***	AJ278507	WRKY transcription factor 1
Reference genes	***GAPDH***	U170005	Glyceraldehyde-3-phosphate dehydrogenase

° = primers analyzed in the third trial.

### High throughput quantitative reverse transcription-polymerase chain reaction (qRT-PCR) conditions

The analysis of gene expression was performed at the Platform for Human and Environmental Genomics, Rennes/France, using a WaferGen SmartChip Real-time PCR system (WaferGen Bio-systems and Takara Bio Inc. USA) with a 5184-well chip. Two extractions per sample were performed, and each extract was deposited in three different wells, for a total of 6 technical replicates per sample. Two samples were analyzed for all 18 genes indicated in Table 1, while the third was run on only a subset of genes, for confirmation purposes.

The qPCR amplifications were performed in 100 nl reaction volume, containing 1 ng/μl cDNA sample, 1x 800/1200 nM primers mix, 1x LightCycler 480 SYBR Green I Master (Roche Diagnostics France), completed with sterile distilled water and dispensed to each well with a SmartChip Multisample Nanodispenser robot. A dilution range with all cDNA was also distributed on the chip in the same conditions. Amplifications were performed in SmartChip Cycler following the same cycling conditions of primer designs. The results were analyzed with SmartChip qPCR Software (v. 2.8.6.1). Wells with faulty amplifications and multiple melting peak curves were excluded. The method of relative quantification adapted from Pfaffl [40] by Thomas *et al* [31], was used to calculate transcript accumulation of genes. qRT-PCR efficiencies were calculated and considered for normalization calculations. The genes out of the criteria were excluded from the analysis (e.g. *WRKY1*). Gene expressions were then normalized with the reference gene *GAPDH*.

### Phytohormone quantification

The phytohormones analyzed were SA, JA, 12-oxophytodienoic acid (OPDA), jasmonoyl-isoleucine (JA-Ile), dihydrojasmonic acid (DHJA) and 1-1-aminocyclopropane carboxylic acid (ACC). This quantification was performed separately on each of the six samples from two independent trials. For each sample, 10 mg (lyophilized material) was treated with an extraction buffer containing 1 ml of 70% methanol, 29% H_2_O, 1% formic acid and internal standards of the phytohormones, isotopically labelled. The labelled standards were included to perform the internal standard quantification, and to allow correction of analytical drifts and to control the extraction and Solid Phase Extraction (SPE) column performance. A second extraction was carried out in 0.5 ml of this same buffer, and supernatants were added to those obtained in the first extraction of the same sample. At each step, samples were stirred and centrifuged to obtain the supernatants, as described for metabolic analysis. Supernatants were evaporated (Turbovap LV, Biotage) and re-suspended in 2% formic acid solution, then purified using an SPE ABN express plate of 30 mg/ml (Biotage). Phytohormones were eluted with methanol, then evaporated and re-suspended in 200 μl of 0.1% formic acid solution. Phytohormones were analyzed by an UHPLC-MS/MS system according to the method described by Maillard *et al*. [41]. The separation and the detection of phytohormones were achieved using a Nexera X2 UHPLC system (Shimadzu, Japan) coupled to a QTrap 6500+mass spectrometer (Sciex, Canada) equipped with an IonDriveTM turbo V electrospray source. Two μl samples were injected into a Kinetex Evo C18 core-shell column (100 x 2.1 mm, 2.6 μm, Phenomenex, USA) at a flow rate of 0.7 ml/min, to phytohormones separation. The column temperature was maintained at 40°C. The mobile phases were composed of solvent A, Milli-Q water and solvent B, acetonitrile LCMS grade (Fisher Optima, UK), both containing 0.1% formic acid (LCMS grade, Fluka analytics, Germany). The analysis was performed in MRM mode, simultaneously, scheduled in positive (5 kV) and in negative (-4.5 kV) modes with a polarity switching of 5 ms. Data analysis was performed using MASSLYNX software (Waters).

### Statistical analyses

The metabolomic data matrix was analyzed with a correlation-based distance calculated with Pearson correlation coefficients and Ward.D2 clustering. A heatmap generated with the “gplot” package in R software version 3.3.3 represented these results. A linear mixed-effects model was fitted by maximum likelihood or restricted maximum likelihood (REML) using with the “lme” package, with significance values set at p ≤0.05. The phytohormones and the metabolites were also analyzed with this test and the results are shown in histogram charts. The *P*. *infestans* biotest data were analyzed with one-way ANOVAs, followed by a Tuckey test (p-value <0.05) for means comparisons.

## Results

### Metabolic profiles in Désirée potato leaves vary according to the elicitor

**Fig 1:**
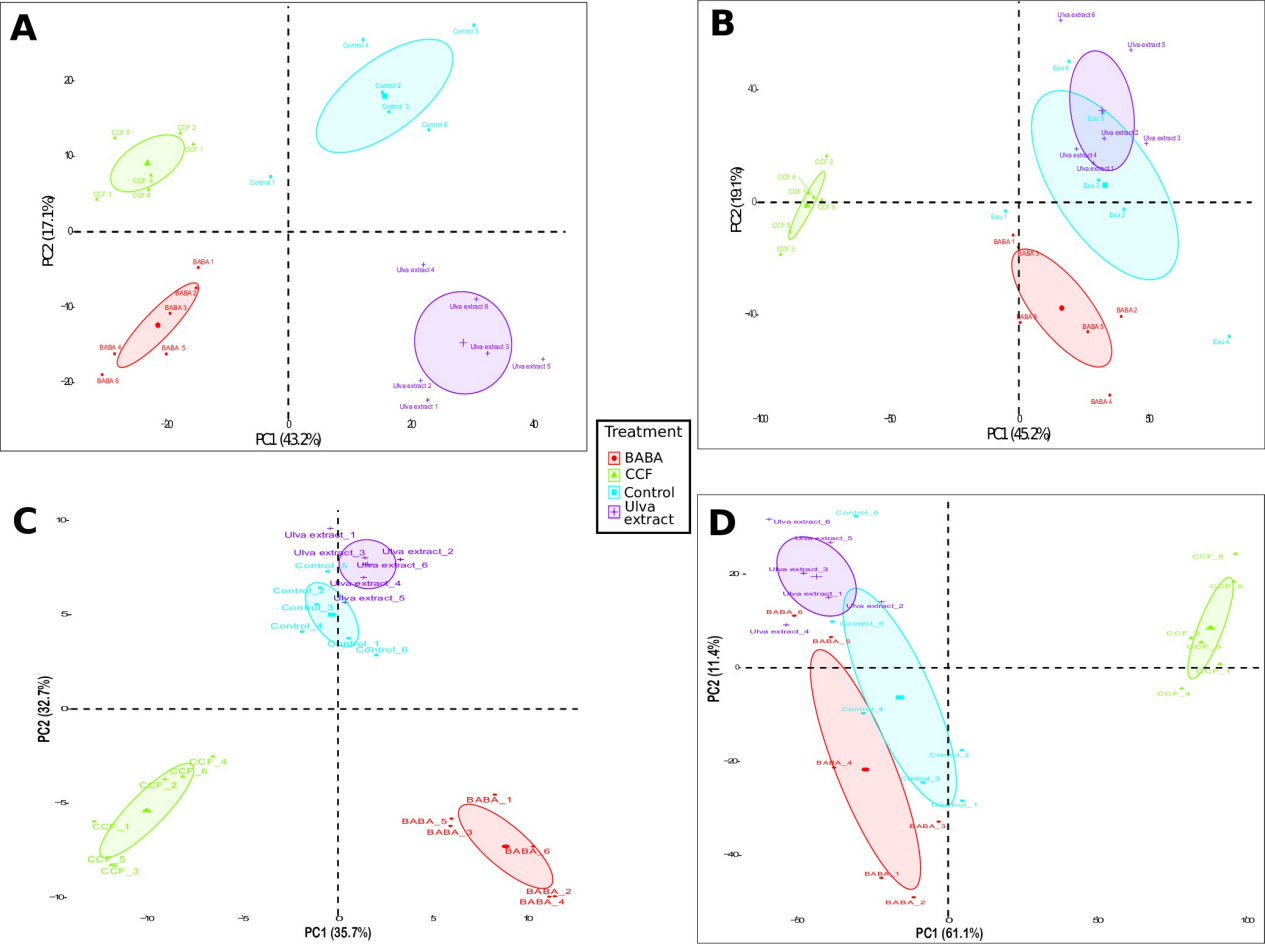
Désirée metabolic profiles of non-supervised PCA on **(A, C)** ESI- and **(B, D)** ESI+ ionization modes in two independent trials. Grouping of individual samples obtained with the various treatments were based on normalized abundances of the identified features from UPLC-qTOF-MS^e^. Each point represents one sample and each color is associated with one treatment: control (blue), CCF (green), BABA (red) and *Ulva* extract (purple). Colored ellipses represent the 95% confidence regions for each treatment.

After filtering with ANOVA (≤ 0.05) tests, fold changes (≥ 2) and q-values (< 0.05) tests, up to 6969 features (1314 from ESI- mode and a further 5655 from ESI+ mode in trial 1; 137 from ESI- mode and 4664 from ESI+ mode in trial 2) were detected in the UPLC-qTOF-MS^e^ analysis of metabolites retrieved from Desirée leaf samples after elicitation with CCF, BABA or *Ulva* extract. For both types of ionization and in each of the two independent trials, four separate clusters corresponding to each treatment were obtained following principal component analysis (PCA) (Fig 1). The combination of axes 1 (PC1) and 2 (PC2) explained respectively 60.3% and 64.3% of the total variance of the ESI- and ESI+ data sets in trial 1 (Fig 1A and 1B), and 68.4% and 72.5% in trial 2 (Fig 1C and 1D). In both cases, the PCA analysis showed that for ESI-, the first axis separated the CCF and BABA clusters from the control and *Ulva* extract clusters. The second axis separated the CCF treatment from the BABA treatments, and the *Ulva* extract from the control (Fig 1A and 1C). For the ESI+, the first axis separated the CCF from the other three treatments, while the second axis separated the *Ulva* extract treatment from the BABA and control treatments (Fig 1B and 1D). Thus, the CCF was well separated from the others treatments.

### Elicitors regulate differentially the metabolic pathways and transcripts involved in defense

**Fig 2:**
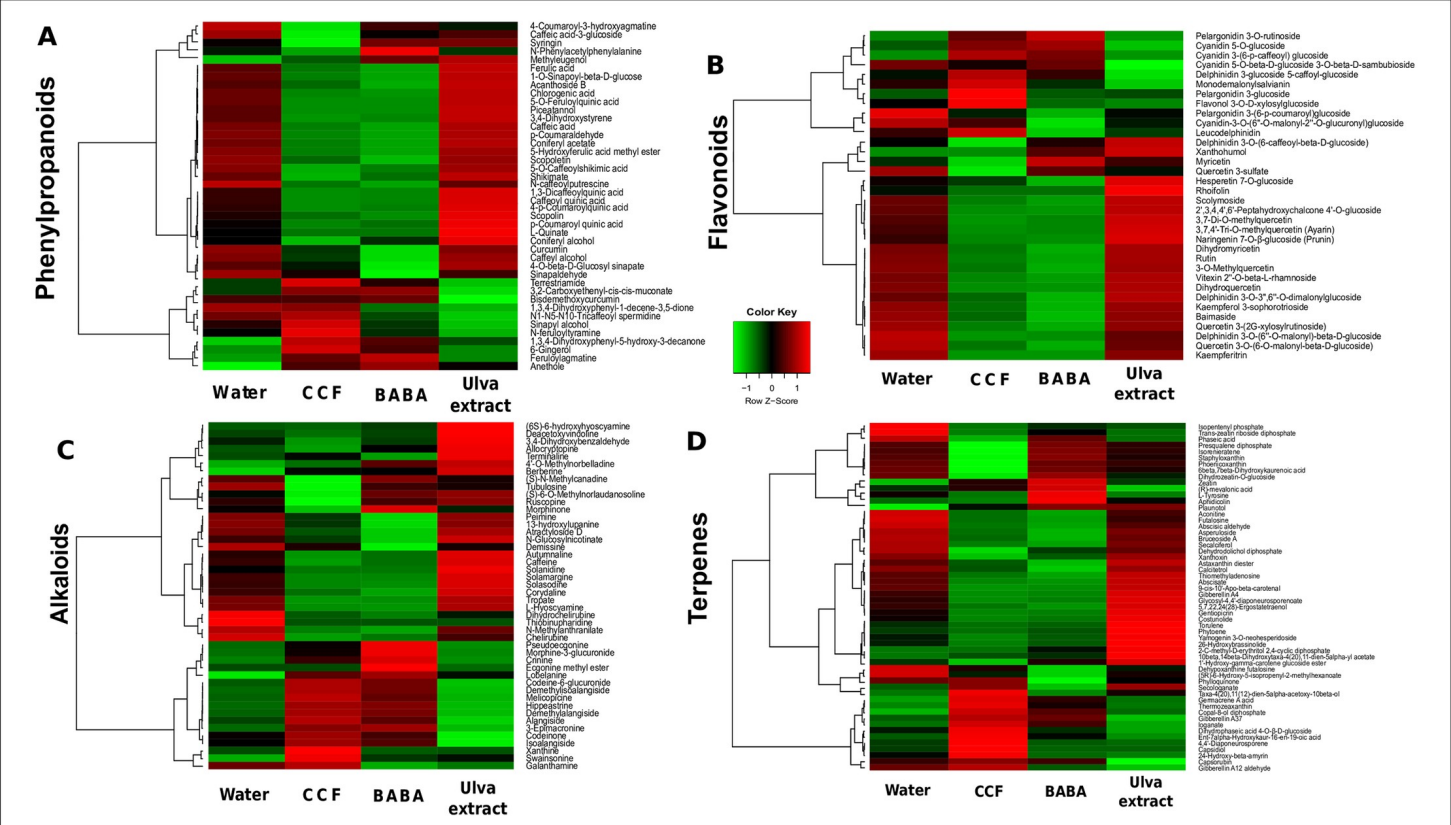
Representation of identified (“Level 2”) metabolites belonging to secondary metabolism pathways. Normalized abundance heatmap of 178 putative secondary metabolites (“Level 2” identification) found in Désirée samples treated with BABA, CCF, *Ulva* extract or water and revealed under ESI- and ESI+ ionization modes: **(A)** 42 phenylpropanoids; **(B)** 34 flavonoids; **(C)** 46 alkaloids; and **(D)** 56 terpenoids. Each row represents one metabolite and the columns represent the average abundance per treatment. The hierarchical clustering tree brings together the metabolites according to the correlation-based distance calculated with Pearson correlation coefficient and Ward linkage. Clusters make it possible to visually identify metabolic profiles specific to each treatment by showing up-regulation (red), neutral effect (black) and down-regulation (green) by treatments.

Due to the lack of available plant specific metabolites resources, we checked the 6969 features detected by ESI- and by ESI+ modes in trial 1 against an in-house database based on public libraries and Solanaceae references and containing 2891 metabolites. These comparisons allowed the identification of 373 putative metabolites (S2 Fig and S1 Table in S1 File), confirmed individually according to predefined criteria. Of these, 64% belonged to four families involved in defense responses against pathogens: 31% were associated with phenylpropanoids and flavonoids (Fig 2A and 2B), 14% with alkaloids (Fig 2C) and 19% with terpenoids (Fig 2D). Other classes of metabolites such as amino acids (14%), lipids (10%), carbohydrates (8%), or nucleotides (2%) were also found.

### Phenylpropanoids and flavonoids

**Fig 3:**
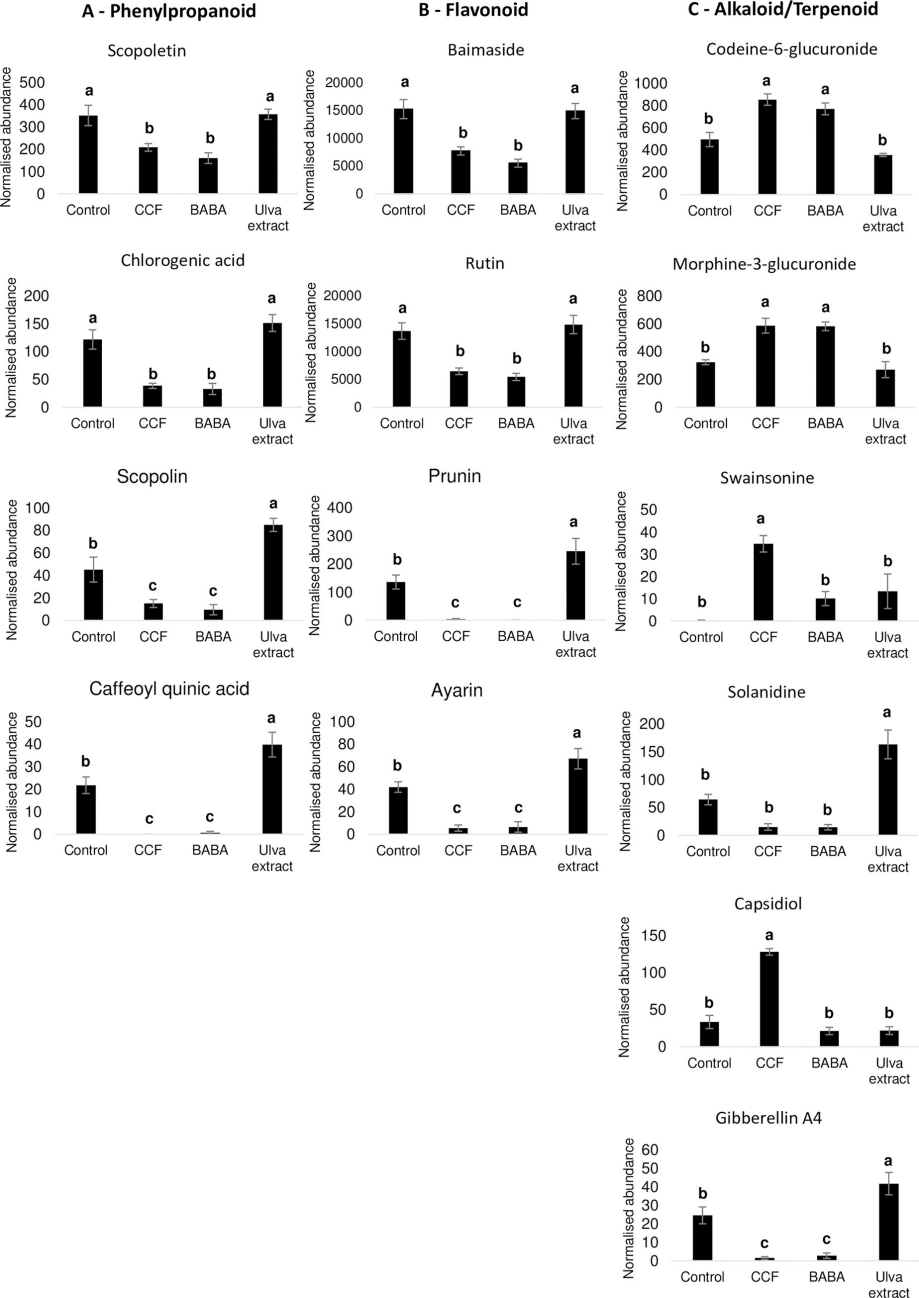
Effect of elicitors on selected metabolite regulations. Semi-quantitative analysis of **(A)** phenylpropanoid, **(B)** flavonoid and **(C)** alkaloid/terpenoid metabolites based on its normalized abundances. Different letters indicate significant differences based on Tukey HSD test (p-value<0.05).

The *Ulva* extract treatment strongly up-regulated, relative to the control (p-value ≤0.05), several antimicrobial metabolites belonging to the phenylpropanoid pathway, such as scopolin and caffeoyl quinic acid. It also tended to increase the contents in scopoletin—the conjugate form of scopolin–and in chlorogenic acid (Fig 3A; S2 Table in S1 File). By contrast, these molecules were significantly down-regulated by CCF and BABA treatments (Fig 3A) compared to the control.

**Fig 4:**
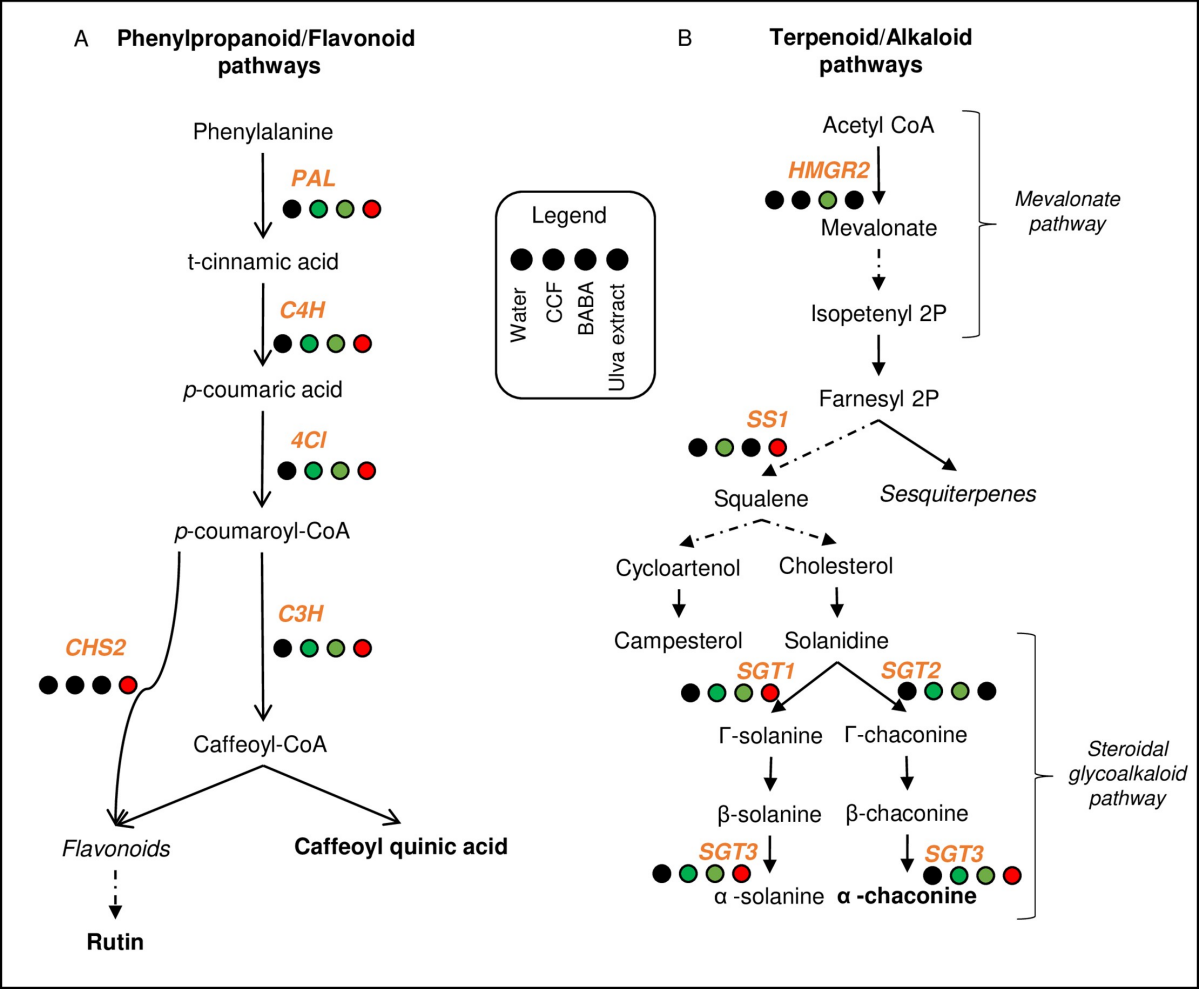
Secondary metabolism pathways and main genes involved in the biosynthesis of the metabolites. Schematic representation of **(A)** phenylpropanoid and flavonoid pathways; **(B)** terpenoid and alkaloid pathways. All pathways were based on KEGG maps. The target genes (orange bold) regulated by the elicitors were analyzed by RT-qPCR. Rutin, caffeoyl quinic acid and α-chaconine (black bold metabolites) were quantified by UPLC-qTOF-MS^e^. Solid arrows indicate a direct link between the metabolites and dotted arrows indicate non-direct link with the mentioned metabolite. The dataset was analyzed by linear mixed-effects model fit by REML and significant differences based on same test (p-value <0.05). The transcript expression relative to the water control for each gene is indicated as colored circles: red circles mean significant induction, green circles significant repression and black circles non-significant differences.

The *Ulva* extract also induced metabolites from the flavonoid pathway, including naringenin 7-O-β-glucoside (prunin) and 3,7,4'-Tri-O-methylquercetin (ayarin) (Fig 3B). As for phenylpropanoids, several major flavonoids, such as rutin and baimaside, were down-regulated after CCF and BABA treatments (Fig 3B). These results were confirmed by the analysis of genes involved in these defense pathways (Fig 4A; S3 and S4A Figs in S1 File). Indeed, the *Ulva* extract treatment up-regulated the phenylpropanoid genes (*PAL*, *C4H* and *4CL*) and the flavonoid gene (*CHS2*) while BABA and CCF down-regulated those genes.

### Alkaloids and terpenoids

Metabolites from the alkaloid pathway, antimicrobial molecules also involved in cell wall reinforcement, such as codeine-6-glucuronide and morphine-3-glucuronide [42], were significantly up-regulated by CCF and BABA treatments compared to the control (Fig 3C; S2 Table in S1 File). By contrast, no treatment modified the regulation of morphinone and codeinone, two precursors of morphine-6-glucuronide and codeine-6-glucuronide respectively. The *Ulva* extract up-regulated solanidine, a sterol glycoalkaloid metabolite, while the other treatments did not affect its production (Fig 3C). RT-qPCR confirmed these regulation patterns with the expression profiles of genes involved in these two pathways (Fig 4B; S5 Fig in S1 File). Indeed, the *Ulva* extract up-regulated the alkaloid gene (*SS1*) and specially the steroidal glycoalkaloid genes *SGT1* and *SGT3* which lead to the production of α-solanine and α-chaconine, respectively. CCF and BABA treatments down-regulated *SGT1*, *SGT2* and *SGT3* (Fig 4B; S4B and S5 Figs in S1 File). Whereas CCF down-regulated *SS1*, BABA down-regulated *HMGR2* (Fig 4B; S5 Fig in S1 File). CCF up-regulated the swainsonine, an alkaloid antimicrobial compound (Fig 3C). Concerning the terpenoid pathway, *Ulva* extract up-regulated gibberellin A4, whereas CCF up-regulated capsidiol, described as a phytoalexin (Fig 3C).

### Primary metabolites

Interestingly, the few metabolites induced only by BABA treatment (UDP-rhamnose, UDP-glucose, GDP-L-glucose from nucleotide sugar metabolism and 7,8-dihydrofolate from the folate pathway) are from the primary metabolism (S6 Fig in S1 File), whereas those specific to the CCF or *Ulva* extract belong to the secondary metabolism.

### *Ulva* extract triggers the JA pathway, while CCF and BABA induce the SA pathway

**Fig 5:**
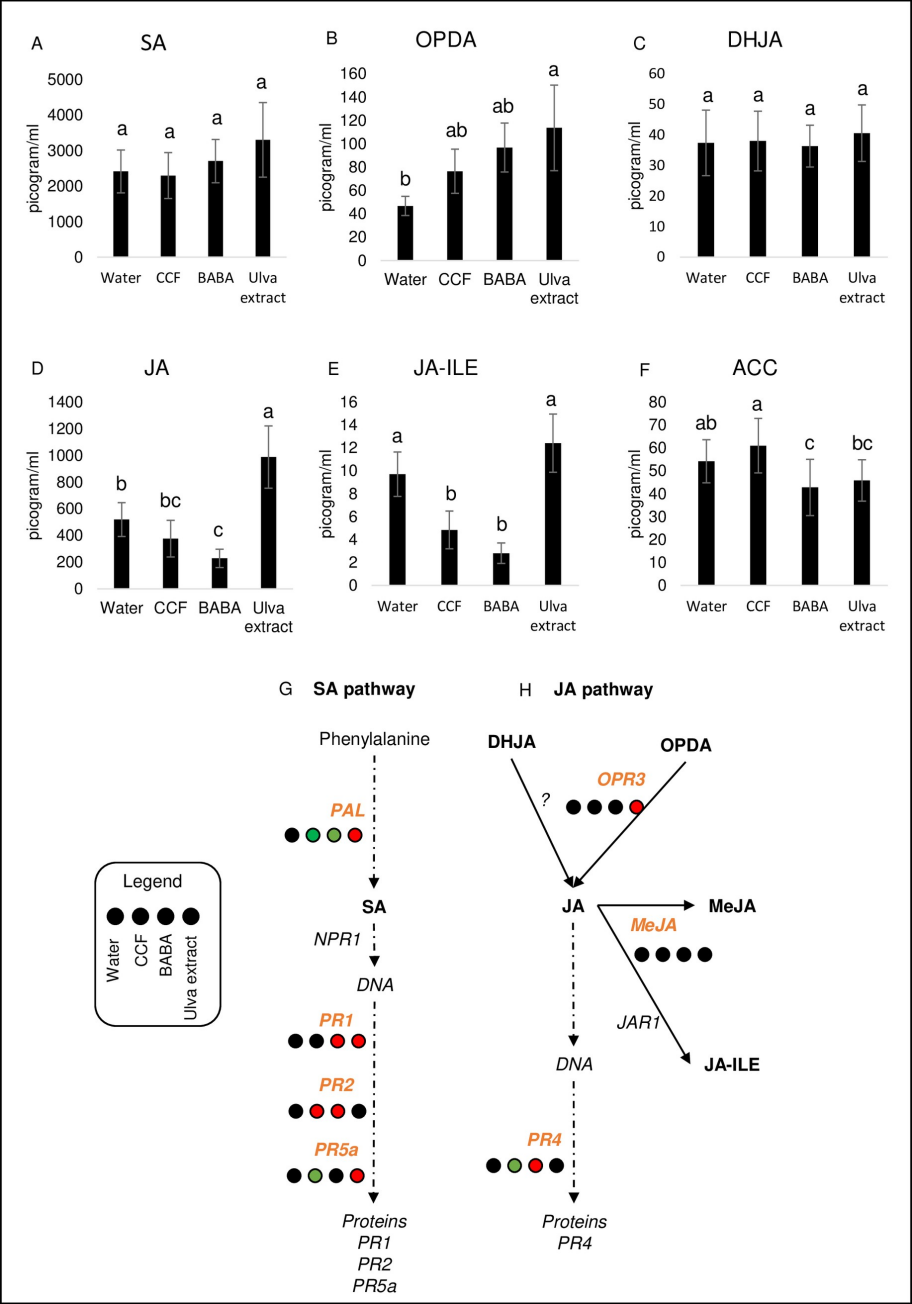
Quantification of phytohormones from secondary metabolism and *PR* gene expression **(A)** SA (p-value 0.3870), **(B)** OPDA (0.1371), **(C)** DHJA (0.9737), **(D)** JA (<0.0001), **(E)** JA-ILE (<0.0001) and **(F)** ACC (0.0032). The target metabolites regulated by the elicitors were analyzed by UHPLC-MS/MS. The concentration in ng/mg of lyophilized material are illustrated by histograms. The target genes related with the phytohormones correspond to either the SA **(G)** or JA **(H)** pathways. The transcript expression relative to the water control for each gene is indicated as colored circles: red circles mean a significant induction, green circles significant repression and black circles non-significant differences. The dataset was analyzed by linear mixed-effects model fit by REML. Different letters indicate significant differences based on same test (p-value <0.05).

The phytohormones were analyzed by using UHPLC-MS/MS coupled to a QTrap 6500+mass spectrometer. The *Ulva* extract significantly (p-value ≤0.01) increased the production of JA and its precursor, the OPDA, compared to the control (Fig 5D and 5B, respectively). It also up-regulated the *OPR3* gene, JA pathway gene (Fig 5H; S7 Fig in S1 File). The concentration of JA-Ile, the active form of JA, failed to increase after *Ulva* extract treatment, but significantly decreased after CCF and BABA treatments (Fig 5E). By contrast, the concentration of DHJA was not modulated by the elicitors (Fig 5C). The expression of the *PR4* gene was up-regulated only by BABA and down-regulated by CCF (Fig 5H; S7 Fig in S1 File). The production of ACC, a precursor of ethylene, was also significantly decreased by BABA compared to control (Fig 5F). For the SA pathway (Fig 5G; S7 Fig in S1 File), the *PR*s genes were regulated by elicitors, even though the production of SA was not modified compared to control (Fig 5A). Indeed, the CCF treatment up-regulated *PR2* and it was the only treatment that down-regulated *PR5a*. BABA treatment up-regulated *PR1* and *PR2* (Fig 5G; S4C and S7 Figs in S1 File) while *Ulva* extract up-regulated *PR2*. As shown previously, *PAL* was up-regulated by *Ulva* extract and down-regulated by CCF and BABA.

### Elicitors differentially induce metabolites effective *in vitro* against *P*. *infestans*

**Fig 6:**
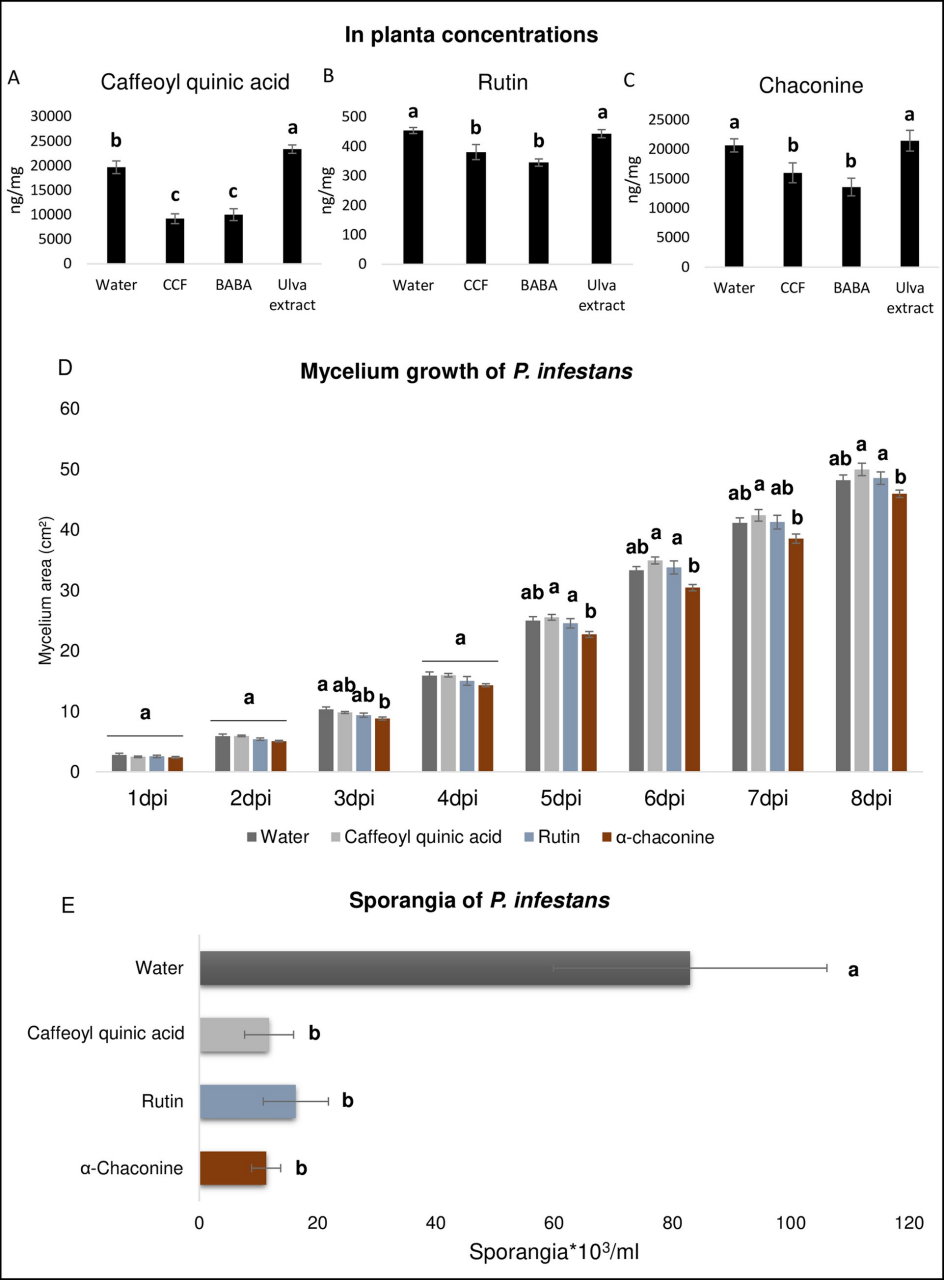
Quantification of secondary antimicrobial metabolites and test *in vitro* against *P*. *infestans*. **(A)** caffeoyl quinic acid, **(B)** rutin and **(C)** α-chaconine, regulated by the elicitors were analyzed by UPLC-qTOF-MS^e^ in ESI- ionization mode. The concentrations in ng/mg are illustrated by histograms. The dataset was analyzed by linear mixed-effects model fit by REML. Different letters indicate significant differences based on same test (p-value <0.05). **(D)** Mycelial growth of *P*. *infestans* was measured for 8 days after deposition of pathogen in Petri dish on pea agar with respectively, α-chaconine (26 μg/ml), rutin (4 μg/ml) or caffeoyl quinic acid (24 μg/ml) or water. ImageJ software was used to this analysis. Different letters indicate significant differences based Tuckey test (p-value <0.05). **(E)** The sporangia production were counted twice by each Petri dish with a Malassez cell.

CCF and BABA treatments significantly (p-value ≤0.05) decreased the concentration of caffeoyl quinic acid, to 9.206 ng/mg and 10.029 ng/mg, respectively, compared to the control (19.692 ng/mg) (Fig 6A). Conversely, the *Ulva* extract increased caffeoyl quinic acid concentration to 23.382 ng/mg (Fig 6A). Similarly, the rutin and the α-chaconine contents were down-regulated by CCF (rutin: 381 ng/mg; and α-chaconine: 16.002 ng/mg) and BABA treatments (rutin: 345 ng/mg; and α-chaconine: 13.579 ng/mg) compared to the control (rutin: 454 ng/mg and α-chaconine: 20.648 ng/mg), but did not change after *Ulva* extract treatment (rutin: 443 ng/mg; p value 0.6428; and α-chaconine: 21.457 ng/mg; p value 0.5591) (Fig 6B and 6C).

The direct effects of these three metabolites against *P*. *infestans* were tested *in vitro* by measuring mycelium growth and sporangia production. Only α-chaconine significantly (p-value ≤0.05) reduced mycelium growth at 3 day post-inoculation (dpi) and from 5 dpi to 8 dpi (Fig 6D). None of the metabolites visibly altered colony morphology. However, all three metabolites significantly reduced the number of sporangia compared to the control (p-value ≤0.01; Fig 6E). The number of sporangia produced in presence of the metabolites was indeed about 6-fold smaller (13.10^3^ sporangia/ml) than in the control medium (70.10^3^ sporangia/ml).

## Discussion

Throughout evolution, plants have developed a capacity to adapt their defense responses according to the stress signals they perceive [3]. As described in the zig-zag model [1], the first layer of plant immunity, PTI, is an essential step in the ability of plants to respond to stresses. However, the level of specificity in induction and nature of the defense pathways constituting PTI is still open to debate. One view, put forward by Jones and Dangl themselves [1] and later supported by Boller and Felix [3], is that PTI is a set of general defense reactions, non-specific of the elicitor that triggered them. Another view, that we used to elaborate our working hypotheses, is that PTI is a mosaic of defense profiles characteristic of the molecular signature of the elicitor that caused them and of the receptors that recognize them (e.g. [43]).

Our data indeed highlighted that the metabolic defense responses of potato to the PAMPs present in CCF markedly differed from those elicited by ‘non-PAMP’ molecules (BABA and *Ulva* extract). Moreover, plant responses to PAMPs fine-tune the interaction between the host and the pathogen producing them by targeting metabolic pathways specific to key functions in pathogen physiology. Together, these data therefore lead to reconsider the diversity of PTI responses, and suggest that responses to PAMPs can explain the specificity component sometimes observed within PTI.

These conclusions are based on three complementary sets of observations. First, **each elicitor treatment induced specific patterns of metabolites but also of genes and phytohormones.** The identification of 373 metabolites, differently induced by the three elicitor preparations, showed that these patterns were predominantly discriminated *via* the secondary metabolism, that represented 64% of metabolites associated with defense pathways, such as phenylpropanoid/flavonoid [44, 45] or alkaloid/terpenoid pathways [42, 46]. This is consistent with the fact that the activation of the immune system (PTI or ETI) triggers a cascade of defense reactions. Early events, such as the liberation of reactive oxygen species (ROS), nitric oxide production and intracellular calcium influx take place within the plant cell wall, to limit infection and spread of pathogens [2]. Intermediate events consist in a signaling cascade *via* the activation of mitogen-activated protein kinases (MAPKs) [47] and phytohormones [48]. Phytohormones such as SA, JA and ethylene are the main mediators of defense responses [49], but auxins, abscisic acid and gibberellins may also be involved [48]. The latest events result from molecular and metabolic re-programming, leading to the production of various defense-related antimicrobial compounds [4]. These compounds are often newly-synthetized molecules, produced to reduce disease symptoms or severity [50]. They may be proteins, such as several PR proteins [51], or secondary metabolites [52, 53] with antimicrobial activities. Most of the metabolites related to the defense responses belong to phenylpropanoid, flavonoid, alkaloid and terpenoid families of compounds [53].

Overall, the *Ulva* extract mainly up-regulated a large set of metabolites from the phenylpropanoid and flavonoid pathways, while CCF and BABA down-regulated them. Within these pathways, a smaller number of metabolites down-regulated by *Ulva* extract treatment were up-regulated by CCF and BABA treatments. The results were less clear for the metabolites belonging to alkaloid and terpenoid, for which each elicitor induced or repressed a different set of metabolites. We also showed that the plants also adapted theirs hormone pathways according to the elicitor. CCF and even more BABA treatments down-regulated both the JA and ethylene pathways. However, CCF and BABA treatments did not induce SA production, although they up-regulated the *PR* genes (*PR1* for BABA and CCF and *PR2* for BABA only) that are SA pathway activation markers. These results confirm and expand previous reports [30, 31, 54, 55], who demonstrated that CCF and BABA can activate the SA signaling pathway. Saubeau *et al*. [30] observed on potato a SA peak 12h after CCF treatment and the *PR*s genes were up-regulated from 24h-48h [31]. In the same way, on soybeans, 15 SA pathway genes are up-regulated 24h after BABA treatment [55]. By contrast, the *Ulva* extract induced the *OPR3* gene and increased OPDA and JA production, as also observed by Jaulneau *et al*. [17] in *Medicago truncatula*. Conversely, in tomato plants, another ulvan of *Ulva lactuca* induced the SA pathway [35], which might suggest an elicitor-dependent effect.

Interestingly, the defense responses induced in the potato by the *Ulva* extract are dominated by phenylpropanoids and flavonoids. This extract up-regulated large-spectrum antimicrobial compounds, such as caffeoyl quinic acid [37] or scopolin [56]. It should be noted that only plants treated with *Ulva* extract showed an over-expression of *PAL*, *C4H* and *4CL* genes, which potentially lead to the production of caffeoyl quinic acid. In addition, the *Ulva* extract up-regulated the gene *CHS2*, the first gene of the flavonoid pathway but this induction was not followed by the production of rutin, a flavonoid antimicrobial compound [29]. The activation of genes from the SGA pathway was also an exclusivity of this treatment. The ratio between the *SGT1* to *SGT2* genes is positively correlated with the ratio between the concentration of solanine and chaconine [57]. Since the treatment with *Ulva* extract did not regulate the *STG2* gene, it can be assumed that the metabolite produced would be α-solanine rather than α-chaconine. This pattern of expression therefore suggests that *Ulva* extract triggers general defense responses that are not specific to any single pathogen.

The response pattern of plants treated with BABA showed that this elicitor mainly targeted the primary metabolism without pathogen inoculation (S5 Fig in S1 File). These results, consistent with those of Pastor *et al*. [19] on *Arabidopsis*, suggest that potato perceives BABA as a primer [20]. Indeed, the benzylisoquinoline alkaloids were up-regulated and the SGA and phenylpropanoid/flavonoid pathway were down-regulated. Other authors reported the induction of phenylpropanoid and flavonoid pathways with the BABA treatment on potato, but only on plants which were subsequently inoculated with the pathogen [25] supporting the role of primer for the BABA.

Second, **the metabolic responses observed with the PAMPs (CCF) target defense pathways that are directly related to *P*. *infestans* biology and fitness**, in two major ways. The first is a depletion of the plant contents in compounds essential for the pathogen, best illustrated with sterol metabolism. Overall, sesquiterpene metabolites are important defense molecules against oomycetes. Indeed, the increase of sesquiterpenes leads to a decrease of sterol and steroid glycoalkaloids (SGA) [26], which are vital for oomycete reproduction [58]. The depletion in sterol contents is probably also linked to the sterol capture by *Phytophthora* elicitins [58].

The other way in which the plant hinders pathogen development is the production of antimicrobial molecules. In our study, only the plants treated with CCF up-regulated capsidiol, belonging to the sesquiterpene pathway. It is of interest to notice that in tobacco, the accumulation of capsidiol combined with the production of ROS increases the resistance to *Phytophthora* spp, and that *P*. *infestans* is highly sensitive to capsidiol [59]. Furthermore, CCF treatment decreased the expression of *SGT1*, *SGT2* and *SGT3* genes. These genes encode the chaconine-related compounds belonging to the SGA [60–62]. Despite the repression of this alkaloid sub-family in plants treated with CCF, other alkaloid metabolites as swainsonine, codeine-6-glucoronide and morphine-3-glucuronide were up-regulated. Interestingly, these molecules all have strong antimicrobial effects. For instance, swainsonine, a phytotoxin produced by endophytic or pathogenic fungi in Fabaceae, disrupts the endomembrane system of the cell, causing irreversible damage [63]. Our observations thus support the results of Yogendra *et al*. [42], who highlighted that codeine-6-glucoronide and morphine-3-glucuronide have antimicrobial properties and a possible role in strengthening the cell wall following *P*. *infestans* infection. In our study, capsidiol and swainsonine were identified through the Cosmos-Level 2 criteria. To our knowledge, neither swainsonine [63] nor capsidiol [64] have been reported in potato. It is not uncommon for metabolomic analyses to detect novel compounds, as shown by the recent identification of codeine or morphine from potato [42]. It would thus be very useful to confirm formally the identification of these molecules by comparison to purified standards.

Furthermore, three metabolites from the target secondary metabolism, rutin, caffeoyl quinic acid and α-chaconine were quantified and tested *in vitro* against *P*. *infestans*. Kröner *et al*. [29] showed that rutin and chlorogenic acid (an isomer of caffeoyl quinic acid) decrease the growth of *P*. *infestans* and *P*. *atrosepticum in vitro*, but that this symptom reduction was only observed with *P*. *atrosepticum* on tuber test. Here, we further showed that these metabolites strongly decrease the sporangia production in *P*. *infestans*, whereas α-chaconine alone decreased the mycelium growth. Our results about α-chaconine match those observed by Fewell and Roddick [65], who showed *in vitro* a reduction by α-chaconine of mycelium growth in *Alternaria brassicicola* and *Phoma medicaginis* [65]. Other studies demonstrated the efficacy of CCF [31] and BABA treatments [25, 33] against *P*. *infestans*. In our study, unexpectedly, CCF and BABA strongly reduced the concentration of these three metabolites, while the *Ulva* extract only increased the concentration of caffeoyl quinic acid. The results of these tests raise the question of the reduction by an elicitor of the amount of efficient metabolites. They suggest that an elicitor is able to influence either negatively or positively the production of metabolites.

One could wonder whether this observation is specific to CCF, or general to PAMPs. Although we had only one elicitor preparation containing PAMPs in our experiments, literature reports in diverse pathosystems suggest that PAMPs indeed often trigger responses specifically designed to restrict infection by the matching pathogen. Gust *et al*. [66] and Wan *et al*. [10] showed that flg22, chitin, LPS or Ef-Tu, *i*.*e*. PAMPs derived from different classes of plant pathogens, regulated different gene patterns. Furthermore, Denoux *et al*. [67] showed that flg22 triggered callose deposition in *A*. *thaliana*, whereas oligogalacturonides did not. Since callose deposition is widely regarded as a cell wall reinforcement response limiting pathogen expansion and cell colonization, this result suggests the induction by PAMPs of an active defense mechanism specific of pathogens.

Third, **CCF induction profiles also highlight the implication of counter-defense mechanisms, and not only of defense responses**. Indeed, our results show that the complex mix of molecular patterns present in CCF induce PTI. Some of the compounds in CCF (notably the sugar fraction) are probably not related to *P*. *infestans* pathogenicity, but can be recognized by the plant and induce defense reactions. Others, notably the elicitins, are directly involved in pathogenicity, as they capture the sterols that are essential for pathogen growth and sporulation [58]. They can thus be regarded as virulence factors. Since they are present in the extracellular medium and get into direct contact with the host (without being secreted in the host cell itself), they can also be used by the plant as a signal for infection and as an elicitor of defense reactions (e.g. [68, 69]). Moreover, following treatment with CCF, the levels of phenylpropanoids and flavonoids, which are actively involved in pathogen restriction [70], are reduced. Therefore, the final defense profile of the plant after CCF application can be regarded as a balance between defense mechanisms through a direct effect on pathogen nutrition and counter-defense through the attenuation of responses induced during PTI.

Since we worked with the culture filtrate only, without inoculating the pathogen, the observed reactions cannot be attributed to ETI, as there was no infectious process involved in our experimental framework. Furthermore, and although elicitins are recognised as virulence/avirulence effectors in *Phytophthora*, the choice of cultivar Désirée, with no known race-specific resistance gene against *P*. *infestans*, further reinforces this conclusion. It supports and extends the report by Peng *et al*. [71] that a filtrate culture of *P*. *parasitica* (including INF1 protein) induces PTI in tomato plants. The elicitins present in CCF may thus have a triple role: sterol quenchers vital for oomycete growth [58], PTI inducers, and virulence/avirulence effectors in race-specific interactions. Other studies with culture filtrate of *Sclerotinia sclerotiorum* [72] also showed the induction of PTI, or basal plant defense. Because of the complex composition of CCF [11], we are not able to determine whether these two antagonistic sets of mechanisms are caused by the same elicitor fraction. However, α–elicitins, which are recognized by the ELR receptor and form a receptor complex with BAK1 [73], are known to trigger several defense mechanisms [74], but could also be involved in counter-defense processes, whereas the sugar fractions of CCF might be more directly responsible for the induction of the general defense pathways.

All our observations were made 48h after application of the elicitors. This choice was made, based on previous results, to focus on the metabolites themselves [30, 31, 37]. However, it probably precluded the assessment of very early events such as oxidative bursts or transient enzyme activities, and does not take into account potential differences between elicitors in uptake/recognition time and half-lives. Therefore, it might be now important to supplement our observations with a time series of observations, to fully encompass the range of defense responses triggered by these elicitors.

The differential induction and diversity of defense patterns according to PAMP/non-PAMP elicitors question the postulated common nature of PTI reactions [1]. This suggests that, contrary to the original assumptions, PTI can be regarded as a heterogeneous set of both general and of pathogen-specific responses, triggered by the molecular signatures of each elicitor, rather than as the common, broad-spectrum responses layer of plant immunity. If this hypothesis is correct, it would explain why some level of specificity occurs within PTI, possibly as a consequence of the pathogen-specific responses resulting from the long co-evolution between host plants and the PAMPs produced by their pathogens. To test the validity of this hypothesis, further studies are now needed: (i) to validate on a larger set of PAMPs and pathosystems the existence of such pathogen-specific responses; (ii) to distinguish the molecular motives responsible for the general and specific types of metabolic responses and; (iii) to untangle the potential pleiotropic effects and interplay between the various molecules eliciting defense reactions in nature. Such studies will bear wide-ranging consequences for plant breeding and managing quantitative resistance. Indeed, PTI potentially provides all plants/crops with an arsenal of antimicrobial compounds, or metabolites, which protects them against pathogens. The high throughput methods now available, such as metabolomics, allow for a reconsideration of our understanding of general and specific defense involved in PTI, and consequently to reconsider the application strategies of elicitors in the field. If each elicitor generates a specific outcome, it might be wise to consider developing elicitor combinations rather than single compounds for high-efficacy and sustainable crop health management.

## Supporting information

S1 File [75]. (PDF)
