# Supplementary material for: A comparison of PTI defense profiles induced in Solanum tuberosum by PAMP and non-PAMP elicitors shows distinct, elicitor-specific responses
Source: PLoS One. 2020 Aug 12;15(8):e0236633. doi: 10.1371/journal.pone.0236633 (PMC7423108; doi:10.1371/journal.pone.0236633)
Supplement: S1 File — (PDF) [file pone.0236633.s001.pdf]

Supporting information

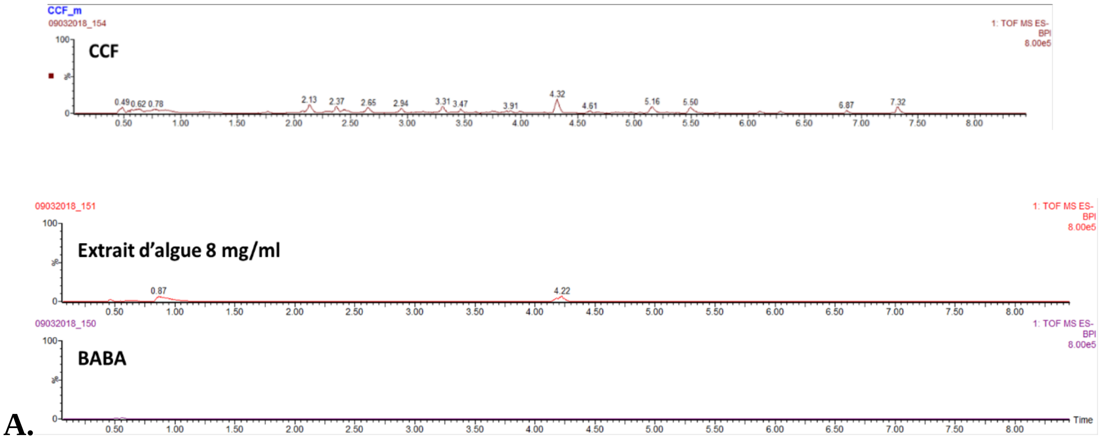

A.

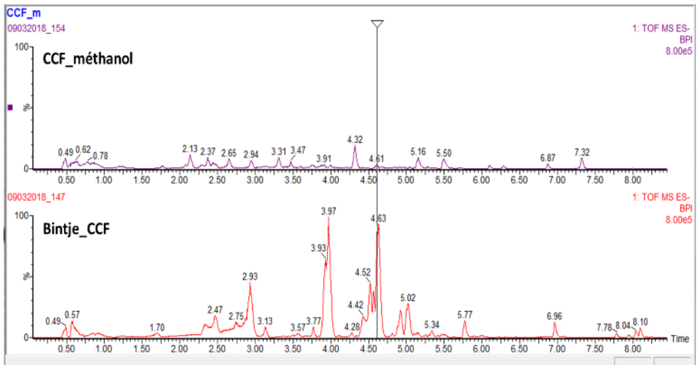

B.

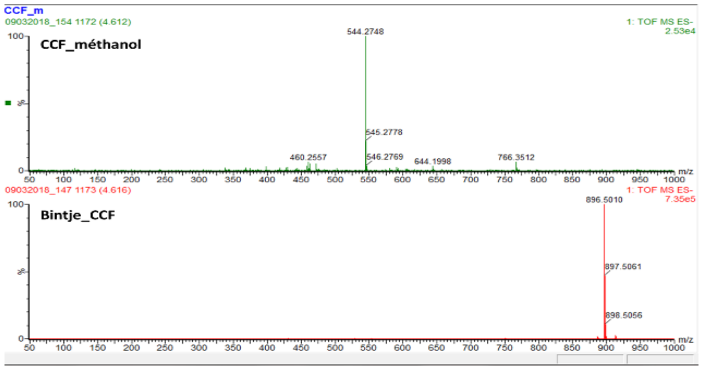

C.

S1 Fig. Elicitor solutions analysis in UPLC-qTOF-MS^e at ESI-. (A) The spectrum of elicitor solutions. These elicitor samples were analysed as the plant samples with the same extraction protocol and UPLC parameters at ESI-. (B-C) The elicitor peaks with retention time closely to plant peaks were verified and no correspondence was found.

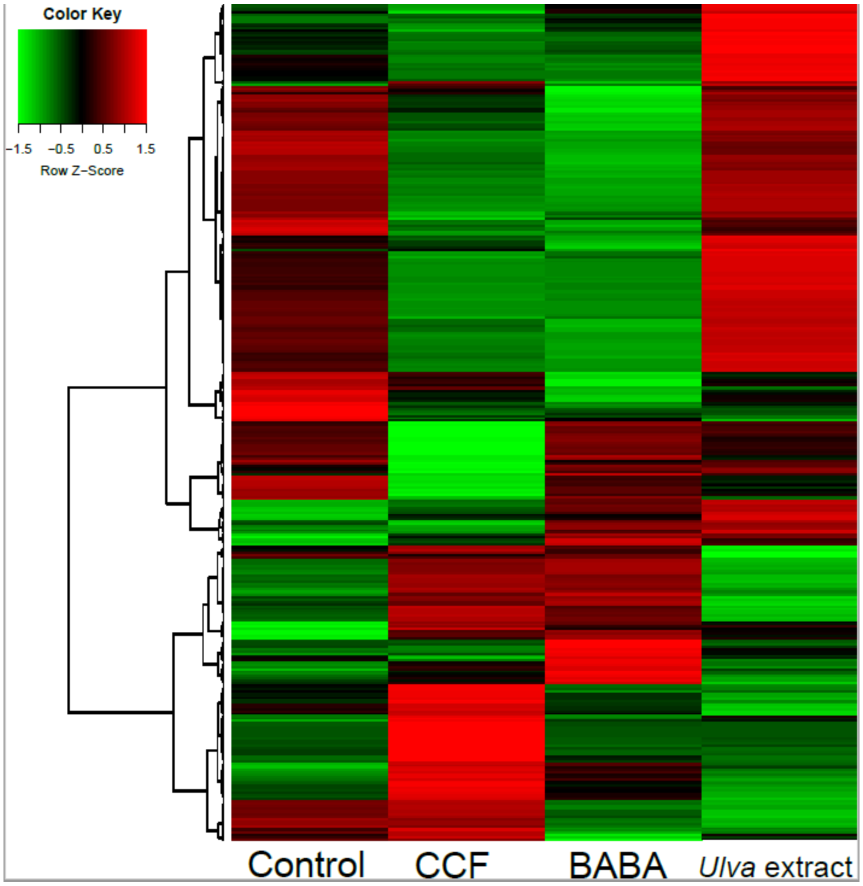

S2 Fig. Désirée metabolic profiles after identification of metabolites. Normalized abundance heatmap of 373 putative identified metabolites ("Level 2" identification) found in Désirée samples treated with BABA, CCF, *Ulva* extract or water and revealed under ESI- and ESI+ ionization modes. Each row represents one metabolite and the columns represent the average abundance per treatment. Associated to the rows, the hierarchical clustering tree brings together the metabolites according to the correlation-based distance calculated with Pearson correlation coefficient and Ward linkage. Clusters make it possible to visually identify metabolic profiles specific to each treatment by showing up-regulation (red), neutral effect (black) and down-regulation (green) by treatments.

S1 Table. Exactly position of the 373 metabolites in Désirée genotype represented by the heatmap. Read from top to bottom. Each metabolite is described by retention time, metabolic unit (m/z), identification, class, chemical formula, error ppm (±5), isotope similarity, p-value (<0.05) and ionization mode.

Position on heatmap	Retention time	m/z	Metabolite	Class	Chemical formula	Error ppm	Isotope Similarity	p-value	Adducts	Ionisation
1	1,87	349,11	Cysteinyldopa	Amino acid	C12H16N2O6S	1,07	92,04	9E-09	M+CH3OH+H	ESI+
2	9,93	633,39	Capsaicin	Amino acid	C18H27NO3	-3,68	80,54	4E-11	2M+Na	ESI+
3	8,28	696,93	Guanosine 3'-diphosphate 5'-triphosphate	Nucleotid	C10H18N5O20P5	-4,72	55,73	4E-07	2M+3H2O+2H	ESI+
4	10,72	882,05	GppppG	Nucleotid	C20H28N10O21P4	1,57	63,64	9E-14	2M+3H2O+2H	ESI+
5	9,65	829,44	17-O-deacetylvindoline	Alkaloid	C23H30N2O5	4,09	84,90	4E-13	2M+H	ESI+
6	9,97	570,03	CDP-4-dehydro-3,6-dideoxy-D-glucose epimer	Carbohydrate	C15H23N3O14P2	-4,55	81,20	0E+00	M+K	ESI+
7	11,68	542,39	Isorenieratene	Terpenoid	C40H48	3,50	81,99	0E+00	2M+3H2O+2H	ESI+
8	2,17	393,13	(S)-N-Methylcanadine	Alkaloid	C21H24NO4+	2,78	83,61	1E-08	M+K	ESI+
9	14,23	841,56	Staphyloxanthin	Terpenoid	C51H78O8	2,68	84,27	2E-09	M+NH4, M+Na, M+K	ESI+
10	9,69	609,31	Presqualene diphosphate	Terpenoid	C30H52O7P2	-0,40	72,68	0E+00	M+Na	ESI+
11	12,75	514,86	Undecaprenyl phosphate alpha-L-Ara4FN	Carbohydrate	C61H100NO8P	-3,29	86,77	5E-15	M+H+Na	ESI+
12	10,63	594,41	Phoenicoxanthin	Terpenoid	C40H52O3	3,12	74,72	1E-09	2M+3H2O+2H	ESI+
13	11,00	397,26	Hercynine	Amino acid	C9H16N3O2	-0,74	95,13	3E-10	2M+H	ESI+
14	11,83	710,50	6beta 7beta-Dihydroxykaurenoic acid	Terpenoid	C20H30O4	-4,07	85,38	2E-15	M+H-2H2O, 2M+ACN+H	ESI+
15	8,63	769,15	MurNAc alpha-1-phosphate	Carbohydrate	C11H20NO11P	1,93	96,36	3E-06	2M+Na	ESI+
16	4,51	300,12	N-caffeoyltyramine	Phenylpropanoid	C17H17NO4	-4,23	77,47	2E-04	M+H	ESI+
17	7,55	401,21	Dihydrozeatin-O-glucoside	Terpenoid	C16H25N5O6	-3,06	88,42	6E-06	M+NH4	ESI+
18	3,05	341,09	Caffeic acid 3-glucoside	Phenylpropanoid	C15H18O9	-2,44	93,10	2E-05	M-H	ESI-
19	9,80	627,08	UDP-alpha-D-ManNAc3NAcA	Carbohydrate	C19H28N4O18P2	4,62	78,01	7E-14	M+H-2H2O	ESI+
20	0,56	351,10	N-Acetyl-L-aspartate	Amino acid	C6H9NO5	-0,64	98,07	9E-04	2M+H	ESI+

21	0,57	395,13	Syringin	Phenylpropanoid	C17H24O9	-3,48	91,19	2E-08	M+Na	ESI+
22	2,93	302,14	S-6-O-Methylnorlaudanosoline	Alkaloid	C17H19NO4	-1,98	93,24	4E-04	M+H	ESI+
23	9,00	651,37	Ruscopine	Alkaloid	C16H26N4O2	0,07	70,50	4E-05	2M+K	ESI+
24	4,90	678,11	Myricetin	Flavonoid	C15H10O8	4,25	83,39	2E-02	2M+ACN+H	ESI+
25	10,70	587,33	4-Coumaroyl-3-hydroxyagmatine	Phenylpropanoid	C14H21N4O3	1,23	86,83	2E-06	2M+H	ESI+
26	14,66	898,09	Malonyl-CoA	Lipid	C24H38N7O19P3S	4,16	86,62	7E-06	M+2Na-H	ESI+
27	8,63	800,15	CoA	Lipid	C21H36N7O16P3S	-1,84	72,40	4E-04	M+CH3OH+H	ESI+
28	12,73	571,38	3-Oxohexadecanoyl-acp	Lipid	C16H29O2S	-4,88	93,35	5E-05	2M+H	ESI+
29	14,99	677,96	3-Iodo-L-tyrosine	Amino acid	C9H10INO3	4,90	94,98	1E-02	2M+ACN+Na	ESI+
30	13,02	311,29	Icosenoic acid	Lipid	C20H38O2	-4,52	90,24	5E-05	M+H	ESI+
31	9,69	860,56	Terpendole B	Terpenoid	C27H35NO3	4,91	74,13	3E-04	2M+NH4	ESI+
32	12,16	828,01	Quercetin 3-sulfate	Flavonoid	C15H10O10S	-2,88	78,77	1E-09	2M+ACN+Na	ESI+
33	11,78	489,29	Tubulosine	Alkaloid	C29H37N3O3	-3,70	87,79	3E-07	2M+3H2O+2H	ESI+
34	9,94	389,25	2-Amino-3-methylvalerate	Amino acid	C6H12NO2-	-3,36	97,15	5E-05	3M-H	ESI-
35	5,26	344,15	Phaseic acid	Terpenoid	C15H20O5	2,34	93,80	7E-03	M+ACN+Na	ESI+
36	8,49	291,20	2R-HPOT	Lipid	C18H30O4	-2,34	86,51	1E-03	M-H2O-H	ESI-
37	3,24	198,11	4-O-Methylnorbelladine	Alkaloid	C10H12O3	0,08	90,10	3E-06	M+NH4	ESI+
38	4,56	721,27	3-Methoxytyramine-betaxanthin	Amino acid	C18H20N2O6	-2,75	84,09	1E-05	2M+H	ESI+
39	5,62	161,10	Methyleugenol	Phenylpropanoid	C11H14O2	-0,43	88,33	2E-04	M+H-H2O	ESI+
40	5,86	758,19	Digalacturonate	Carbohydrate	C12H18O13	3,91	58,88	1E-06	2M+NH4	ESI+
41	7,49	731,34	Digalactosylceramide sulfate	Lipid	C31H56NO16S	-3,16	78,75	6E-06	M+H	ESI+
42	6,01	673,25	Berberine	Alkaloid	C20H18NO4+	-1,50	94,39	9E-03	2M+H	ESI+
43	7,23	551,28	Feruloylputrescine	Phenylpropanoid	C14H20N2O3	-4,76	86,50	3E-02	2M+Na	ESI+
44	8,49	291,20	9S-HPOT	Lipid	C18H30O4	-2,34	86,51	1E-03	M-H2O-H	ESI-
45	0,54	346,05	AMP	Carbohydrate	C10H14N5O7P	-3,35	94,37	1E-04	M-H	ESI-
46	0,86	346,05	N-Acetyl-D-glucosamine 6-phosphate	Carbohydrate	C8H16NO9P	-0,04	89,29	1E-03	M+FA-H	ESI-
47	0,74	306,08	glutathione	Amino acid	C10H17N3O6S	-2,91	91,36	8E-04	M-H	ESI-

48	2,61	277,17	p-Coumaroylagmatine	Phenylpropanoid	C14H20N4O2	-1,74	98,71	4E-03	M+H	ESI+
49	0,55	482,11	2-succinatobenzoate	Terpenoid	C11H8O5-2	-4,49	97,26	5E-04	2M+ACN+H	ESI+
50	4,32	399,12	Xanthohumol	Flavonoid	C21H22O5	-0,52	89,48	2E-13	M+2Na-H	ESI+
51	8,49	291,20	12-OPDA	Lipid	C18H28O3	-2,37	86,74	1E-03	M-H	ESI-
52	11,47	339,29	plaunotol	Terpenoid	C20H34O2	-3,11	94,43	2E-03	M+CH3OH+H	ESI+
53	0,72	173,14	N-Carbamoylputrescine	Phenylpropanoid	C5H13N3O	-1,31	94,24	2E-03	M+ACN+H	ESI+
54	9,34	524,36	trans-Tetradec-2-enoyl-acp	Lipid	C14H25OS	1,31	81,83	1E-02	2M+ACN+H	ESI+
55	8,12	358,37	Behenic acid	Lipid	C22H44O2	-3,78	95,82	4E-02	M+NH4	ESI+
56	5,24	777,28	Allocryptopine	Alkaloid	C21H23NO5	-4,37	94,91	4E-04	2M+K	ESI+
57	5,50	383,13	7-deoxyloganic acid	Terpenoid	C16H24O9	1,27	84,91	9E-03	M+Na, 2M+ACN+H	ESI+
58	11,67	708,47	1-Hydroxy-gamma-carotene glucoside ester	Terpenoid	C47H67O7	-0,12	70,68	7E-06	M+H-2H2O	ESI+
59	2,78	660,17	Delphinidin 3-O-6-caffeoyl-beta-D-glucoside	Flavonoid	C30H27O15+	0,20	95,76	1E-03	M+CH3OH+H	ESI+
60	5,28	649,29	6S-6-hydroxyhyoscyamine	Alkaloid	C17H23NO4	-1,13	94,33	1E-02	2M+K	ESI+
61	11,74	401,21	10beta 14beta-Dihydroxytaxa-420 11-dien-5alpha-yl acetate	Terpenoid	C22H34O4	1,65	91,99	8E-03	M+K	ESI+
62	11,87	559,31	3-Oxotetradecanoyl-acp	Lipid	C14H25O2S	-3,50	83,81	1E-04	2M+FA-H	ESI-
63	1,06	276,99	2-C-methyl-D-erythritol 2 4-cyclic diphosphate	Terpenoid	C5H12O9P2	-3,68	92,60	2E-08	M-H	ESI-
64	2,00	299,08	Salicylate &beta-D-glucose ester	Phenylpropanoid	C13H16O8	-2,76	85,38	1E-07	M-H	ESI-
65	2,01	137,02	3 4-Dihydroxybenzaldehyde	Alkaloid	C7H6O3	-2,28	92,06	3E-07	M-H	ESI+
66	4,26	207,14	Methyl jasmonate	Lipid	C13H20O3	-1,56	89,53	3E-02	M+H-H2O	ESI+
67	3,24	198,11	Coniferyl alcohol	Phenylpropanoid	C10H12O3	0,08	90,10	3E-06	M+NH4	ESI+
68	0,54	355,09	D-glucono-1 5-lactone	Carbohydrate	C6H10O6	-3,93	96,66	2E-08	2M-H	ESI-
69	10,27	579,39	Torulene	Terpenoid	C40H54	-4,84	88,38	1E-03	M+2Na-H	ESI+
70	5,39	559,14	Rhoifolin	Flavonoid	C27H30O14	-1,62	75,17	2E-07	M-H2O-H	ESI-
71	10,49	703,40	Isonuatigenin 3-rhamnosyl-1- >2-glucoside	Flavonoid	C39H62O13	-0,96	96,67	2E-04	M+H-2H2O	ESI+
72	0,76	341,11	Cellobiose	Carbohydrate	C12H22O11	-2,91	85,39	4E-06	M-H	ESI-

73	10,50	745,41	Yamogenin 3-O-neohesperidoside	Terpenoid	C39H62O12	-1,42	90,31	1E-03	M+Na	ESI+
74	3,17	261,17	Traumatic acid	Lipid	C12H20O4	3,09	96,81	6E-07	M+CH3OH+H	ESI+
75	10,32	461,32	26-Hydroxybrassinolide	Terpenoid	C28H48O7	-3,34	92,61	1E-02	M+H-2H2O	ESI+
76	11,12	797,44	deacetoxyvindoline	Alkaloid	C23H30N2O4	-4,59	83,05	5E-04	2M+H	ESI+
77	12,37	589,47	Phytoene	Terpenoid	C40H64	2,82	80,38	7E-05	M+2Na-H	ESI+
78	2,98	271,10	Vestitol	Flavonoid	C16H16O4	-3,45	83,58	2E-05	M-H	ESI-
79	3,39	337,09	Gentiopicrin	Terpenoid	C16H18O8	-2,84	94,46	3E-07	M-H	ESI-
80	3,62	735,21	p-Coumaroyl quinic acid	Phenylpropanoid	C16H18O8	-3,26	97,00	7E-12	2M+Hac-H	ESI-
81	12,32	465,30	Costunolide	Terpenoid	C15H20O2	1,33	94,53	6E-03	2M+H	ESI+
82	3,49	335,08	Scopolin	Phenylpropanoid	C16H18O9	-3,23	82,90	1E-05	M-H2O-H	ESI-
83	3,42	339,11	2-Isopropylmaleate	Amino acid	C7H10O4	0,93	88,63	1E-05	2M+Na	ESI+
84	3,00	229,10	Formyl-N-acetyl-5-methoxykynurenamine	Amino acid	C13H16N2O4	-1,73	93,54	5E-03	M+H-2H2O	ESI+
85	4,65	191,06	L-Quinate	Amino acid	C7H12O6	-4,66	91,28	2E-05	M-H	ESI-
86	8,87	398,34	Solanidine	Alkaloid	C27H43NO	-3,81	90,26	6E-04	M+H	ESI+
87	4,63	499,12	Formononetin 7-O-glucoside-6-O-malonate	Flavonoid	C25H24O12	-2,13	95,10	2E-04	M+H-H2O, M+Na	ESI+
88	4,46	513,10	Syringetin 3-rhamnoside	Flavonoid	C23H24O12	2,35	77,43	1E-03	M+Na-2H	ESI-
89	11,02	488,33	Sphingosyl-phosphocholine	Lipid	C23H50N2O5P	-1,84	76,64	5E-02	M+Na	ESI+
90	3,43	375,13	Secologanate	Terpenoid	C16H22O10	2,81	92,40	2E-02	M+H	ESI+
91	13,43	607,29	Euphornin	Terpenoid	C33H44O9	4,53	84,07	3E-02	M+Na	ESI+
92	3,33	191,11	Tuberonic Acide	Hormone	C12H18O4	-1,57	89,68	4E-04	M+H-2H2O	ESI+
93	5,56	417,15	Sinapaldehyde	Phenylpropanoid	C11H12O4	-4,55	94,03	6E-03	2M+H	ESI+
94	2,49	589,17	Guanosine	Nucleotid	C10H13N5O5	-2,15	98,14	4E-03	2M+Na	ESI+
95	3,27	137,10	9-Oxononanoic acid	Lipid	C9H16O3	-3,14	90,01	4E-04	M+H-2H2O	ESI+
96	4,73	471,16	Abscisic acid glucose ester	Terpenoid	C21H30O9	4,63	77,83	2E-02	M+2Na-H	ESI+
97	5,03	351,12	Curcumin	Phenylpropanoid	C21H20O6	-3,34	95,30	9E-03	M+H-H2O	ESI+
98	3,88	485,11	Malonyldaidzin	Flavonoid	C24H22O12	1,52	93,60	1E-02	M+H-H2O	ESI+

99	4,19	414,33	Peimine	Alkaloid	C27H45NO3	-4,91	87,23	4E-03	M+H-H2O, M+H	ESI+
100	5,61	592,38	13-hydroxylupanine	Alkaloid	C15H24N2O2	-3,98	62,57	3E-03	2M+ACN+Na	ESI+
101	4,45	165,05	Caffeyl alcohol	Phenylpropanoid	C9H10O3	-4,92	90,07	1E-04	M-H	ESI-
102	4,59	385,19	12-Hydroxyjasmonic acid 12-O-ß-D-glucoside	Lipid	C19H30O8	-3,23	79,70	1E-02	M-H	ESI-
103	3,44	385,11	4-O-beta-D-Glucosyl-sinapate	Flavonoid	C17H22O10	-4,02	81,12	2E-04	M-H	ESI-
104	6,06	543,28	Atractyloside D	Alkaloid	C27H46O12	-1,84	74,66	3E-05	M-H2O-H	ESI-
105	3,20	541,16	D-Lombricine	Amino acid	C6H15N4O6P	4,92	63,01	2E-05	2M+H	ESI+
106	3,68	771,21	Portulacaxanthin II	amino acid	C18H18N2O7	-3,77	90,97	3E-03	2M+Na	ESI+
107	5,85	415,32	Calcitetrol	Terpenoid	C27H44O4	-4,81	93,43	3E-03	M+H-H2O	ESI+
108	3,60	292,99	5-3-carboxy-3-oxopropyl-4 6-dihydroxypicolinate	Amino acid	C10H8NO7	4,00	94,86	7E-03	M+K	ESI+
109	2,82	357,08	DCDC	Amino acid	C7H8O4	-0,86	92,80	1E-03	2M+FA-H	ESI-
110	2,15	203,08	L-Tryptophan	Amino acid	C11H12N2O2	-3,14	97,23	8E-06	M-H	ESI-
111	4,42	469,33	Rishitinol	Alkaloid	C15H22O2	-3,70	96,44	7E-03	2M+H	ESI+
112	3,95	189,07	4-Guanidinobutanamide	Amino acid	C5H12N4O	1,36	96,89	2E-02	M+2Na-H	ESI+
113	5,34	381,31	Secalciferol	Terpenoid	C27H44O3	-4,67	95,36	2E-03	M+H-2H2O	ESI+
114	3,82	741,19	Quercetin 3-2G-xylosylrutinoside	Flavonoid	C32H38O20	-1,45	96,25	2E-07	M-H	ESI-
115	3,33	297,11	p-Coumaraldehyde	Phenylpropanoid	C9H8O2	-3,62	90,29	4E-03	2M+H	ESI+
116	5,49	559,14	Kaempferitrin	Flavonoid	C27H30O14	-2,06	78,63	1E-07	M-H2O-H	ESI-
117	4,46	491,12	Iristectorigenin A 7-O-glucoside	Flavonoid	C23H24O12	-1,73	92,46	2E-07	M-H	ESI-
118	3,51	303,13	N-Methylanthranilate	Alkaloid	C8H9NO2	-3,58	90,92	2E-05	2M+H	ESI+
119	6,86	539,22	N-caffeoylputrescine	Phenylpropanoid	C13H18N2O3	-4,80	91,64	5E-03	2M+K	ESI+
120	3,37	277,15	4-Hydroxyphenylethanol	Amino acid	C8H10O2	1,19	91,54	8E-03	M+H-2H2O, 2M+H	ESI+
121	3,90	551,10	Quercetin 3-O-6-O-malonyl-beta-D-glucoside	Flavonoid	C24H22O15	-2,14	85,00	5E-06	M+H	ESI+
122	5,32	727,24	Bruceoside A	Terpenoid	C32H42O16	-3,22	75,14	3E-06	M+FA-H	ESI-
123	4,03	727,08	XMP	Nucleotid	C10H13N4O9P	4,83	78,52	1E-05	2M-H	ESI-

124	3,62	175,04	Scopoletin	Phenylpropanoid	C10H8O4	-1,06	95,46	1E-04	M+H-H2O	ESI+
125	3,66	649,14	Baimaside	Flavonoid	C27H30O17	-2,23	96,55	8E-05	M+H, M+Na, M+K	ESI+
126	3,68	271,02	Sedoheptulose 7-phosphate	Carbohydrate	C7H15O10P	4,77	90,81	3E-05	M-H2O-H	ESI-
127	3,91	667,15	Phosphatidylcholine	Lipid	C10H18NO8P	-3,26	92,67	1E-04	2M+FA-H	ESI-
128	3,46	497,16	6-Pyruvoyltetrahydropterin	Folate	C9H11N5O3	0,01	90,66	4E-02	2M+Na	ESI+
129	6,31	435,09	Asperuloside	Terpenoid	C18H22O11	3,11	83,56	1E-05	M+Na-2H	ESI-
130	2,83	465,10	5-Hydroxyferulic acid methyl ester	Phenylpropanoid	C10H10O5	-2,31	95,50	1E-05	2M+FA-H	ESI-
131	4,38	317,06	3-O-Methylquercetin	Flavonoid	C16H12O7	-2,74	99,39	1E-05	M+H	ESI+
132	5,19	690,45	3-Oxooctadecanoyl-acp	Lipid	C18H33O2S	-3,97	91,58	2E-04	2M+ACN+Na	ESI+
133	4,65	513.1025	Maackiain-3-O-glucosyl-6-O-malonate	Flavonoid	C25H24O13	-2,36	75,60	4E-10	M-H2O-H	ESI-
134	1,90	476,12	Indolylmethylthiohydroximate	Amino acid	C10H10N2OS	4,11	93,94	3E-04	2M+ACN+Na	ESI+
135	4,88	521,13	Kanokoside A	Alkaloid	C21H32O12	-3,01	91,99	1E-06	M+H-2H2O, M+2Na-H	ESI+
136	2,54	179,03	Caffeic acid	Phenylpropanoid	C9H8O4	-4,99	89,89	3E-04	M-H	ESI-
137	3,51	303,05	Dihydromyricetin	Flavonoid	C15H12O8	0,16	99,68	1E-04	M+H-H2O	ESI+
138	4,03	609,15	Rutin	Flavonoid	C27H30O16	0,13	98,36	1E-06	M-H	ESI-
139	2,30	254,17	Traumatin	Lipid	C12H20O3	-3,75	94,51	1E-03	M+ACN+H	ESI+
140	2,98	264,12	Coniferyl acetate	Phenylpropanoid	C12H14O4	-3,48	97,49	7E-07	M+ACN+H	ESI+
141	2,83	303,05	Dihydroquercetin	Flavonoid	C15H12O7	-3,55	84,98	6E-06	M-H	ESI-
142	3,06	595,17	Butein 3 2-diglucoside	Flavonoid	C27H32O15	-1,99	77,83	2E-07	M-H	ESI-
143	5,08	559,14	Vitexin 2-O-beta-L-rhamnoside	Flavonoid	C27H30O14	-1,49	92,99	7E-05	M-H2O-H	ESI-
144	2,44	353,09	Fraxin	Phenylpropanoid	C16H18O10	-1,45	78,96	5E-06	M+H-H2O	ESI+
145	2,87	539,14	2-Methyl-1-hydroxypropyl-ThPP	Amino acid	C16H27N4O8P2S	-1,28	88,65	3E-05	M+ACN+H	ESI+
146	3,19	254,15	L-Hyoscyamine	Alkaloid	C17H23NO3	-3,48	94,39	1E-02	M+H-2H2O	ESI+
147	2,96	148,05	tropate	Alkaloid	C9H9O3	-3,31	90,08	4E-03	M+H-H2O	ESI+
148	2,64	426,02	GDP	Nucleotid	C10H15N5O11P2	3,32	96,45	2E-02	M+H-H2O	ESI+
149	5,07	539,27	Xanthoxin	Terpenoid	C15H22O3	-4,07	96,32	3E-05	M+H, 2M+K	ESI+

150	2,98	357,06	5-O-Caffeoylshikimic acid	Phenylpropanoid	C16H16O8	4,92	86,05	4E-05	M+Na-2H	ESI-
151	5,84	553,23	Dehydrodolichol diphosphate	Terpenoid	C25H44O7P2	-0,66	74,80	2E-04	M+Cl	ESI-
152	5,31	644,31	Aconitine	Terpenoid	C34H47NO11	3,00	67,82	8E-03	M-H	ESI-
153	5,74	293,14	Abscisic aldehyde	Terpenoid	C15H20O3	-4,56	84,29	6E-08	M+FA-H	ESI-
154	2,52	353,09	1-O-Vanilloyl-beta-D-glucose	Phenylpropanoid	C14H18O9	4,60	95,48	1E-04	M+Na	ESI+
155	6,31	435,09	Futalosine	Terpenoid	C19H18N4O7	-0,03	82,29	1E-05	M+Na-2H	ESI-
156	3,60	866,16	2-Methylacetoacetyl-CoA	Amino acid	C26H42N7O18P3S	-2,92	66,65	4E-04	M+H	ESI+
157	3,34	769,20	Chelirubine	Alkaloid	C21H16NO5	-1,41	89,67	3E-06	2M+FA-H	ESI-
158	2,47	516,09	Delphinidin 3-O-6-O-malonyl-beta-D-glucoside	Flavonoid	C24H23O15	3,14	86,95	1E-02	M+H-2H2O	ESI+
159	4,99	387,14	2-Descarboxy-betanidin	Amino acid	C17H17N2O6	2,12	96,26	1E-02	M+ACN+H	ESI+
160	6,96	663,33	norajmaline	Alkaloid	C19H24N2O2	3,38	96,14	3E-02	2M+K	ESI+
161	4,49	429,12	Hesperetin 7-O-glucoside	Flavonoid	C22H24O11	4,84	86,28	2E-02	M+H-2H2O	ESI+
162	2,66	367,10	L-Fucose	Carbohydrate	C6H12O5	-4,30	97,09	2E-03	2M+K	ESI+
163	2,92	319,13	N-Glucosylnicotinate	Alkaloid	C12H16NO7	3,82	88,89	6E-03	M+CH3OH+H	ESI+
164	5,01	307,26	Stearate	Lipid	C18H36O2	1,89	90,45	1E-02	M+Na	ESI+
165	7,36	364,32	Terminaline	Alkaloid	C23H41NO2	-3,62	98,07	3E-02	M+H	ESI+
166	3,00	215,06	Caffeine	Phenylpropanoid	C8H10N4O2	1,34	93,40	2E-06	M+Na-2H	ESI-
167	6,22	747,39	Autumnaline	Alkaloid	C21H27NO5	4,86	90,93	5E-02	2M+H	ESI+
168	4,81	397,15	7-deoxyloganin	Terpenoid	C17H26O9	-2,21	92,07	2E-03	M+Na	ESI+
169	3,21	353,14	Gibberellin A4	Terpenoid	C19H24O5	1,76	80,33	2E-06	M+Na-2H	ESI-
170	2,79	719,16	Rosmarinate	Amino acid	C18H16O8	-3,32	97,62	8E-08	2M-H	ESI-
171	4,64	515,12	1 3-Dicaffeoylquinic acid	Phenylpropanoid	C25H24O12	-1,05	95,01	6E-07	M-H	ESI-
172	3,68	707,18	Caffeoyl quinic acid	Phenylpropanoid	C16H18O9	-4,68	86,27	7E-12	2M-H	ESI-
173	3,02	179,03	3-3 5-Dihydroxyphenyl-2-propenoic acid	Phenylpropanoid	C9H8O4	-3,47	96,73	3E-06	M-H	ESI-
174	2,98	163,04	4-Hydroxycoumarin	Phenylpropanoid	C9H6O3	3,06	98,95	3E-05	M+H-H2O, M+H, M+ACN+H	ESI+

175	3,00	513,10	Malonylglycitin	Flavonoid	C25H24O13	-2,33	75,47	1E-04	M-H2O-H	ESI-
176	3,25	343,08	3 7 4-Tri-O-methylquercetin Ayarin	Flavonoid	C18H16O7	-3,13	83,98	2E-05	M-H	ESI-
177	4,36	433,11	Naringenin 7-O-ß-glucoside =Prunin	Flavonoid	C21H22O10	-2,28	89,65	5E-10	M-H	ESI-
178	4,20	850,49	Solamargine	Alkaloid	C45H73NO15	-0,67	99,19	9E-04	M+H-H2O	ESI+
179	4,58	414,34	Solasodine	Alkaloid	C27H43NO2	-2,77	97,31	7E-04	M+H	ESI+
180	0,61	335,07	3R 4R-3 4-Dihydroxycyclohexa-1 5-diene-1-carboxylate	Amino acid	C7H8O4	-4,29	94,58	6E-04	2M+Na	ESI+
181	3,26	337,09	4-p-Coumaroylquinic acid	Phenylpropanoid	C16H18O8	-2,87	93,21	8E-07	M-H	ESI-
182	6,15	617,35	Glycosyl-4 4-diaponeurosporenoate	Terpenoid	C36H50O7	4,36	76,18	6E-04	M+Na	ESI+
183	2,90	833,23	Daidzin	Flavonoid	C21H20O9	0,55	95,46	1E-13	2M+H	ESI+
184	5,06	583,14	Arbutin	Carbohydrate	C12H16O7	-2,76	94,30	2E-09	2M+K	ESI+
185	0,51	314,08	Dihydrosanguinarine	Alkaloid	C20H15NO4	2,77	79,45	2E-03	M-H2O-H	ESI-
186	6,08	719,16	3 7-Di-O-methylquercetin	Flavonoid	C17H14O7	0,27	91,21	3E-02	2M+Hac-H	ESI-
187	4,17	587,19	100-1	Terpenoid	C31H34O10	-1,73	71,43	1E-02	M+Na-2H	ESI-
188	3,21	161,02	5-Amino-4-imidazolecarboxyamide	Nucleotid	C4H6N4O	-0,06	94,19	2E-04	M+Cl	ESI-
189	4,51	394,31	9-cis-10-Apo-beta-carotenal	Terpenoid	C27H36O	-3,70	84,46	5E-03	M+NH4	ESI+
190	5,18	529,13	Luteolin 7-glucoside-4-Z-2-methyl 1-2-butenoate	Flavonoid	C26H26O12	-2,27	77,08	4E-06	M-H	ESI-
191	2,86	555,11	CDP-ribitol	Carbohydrate	C14H25N3O15P2	-1,47	90,93	2E-05	M+NH4	ESI+
192	3,47	460,12	5-Butyrylphosphoinosine	Nucleotid	C14H19N4O9P	3,32	96,82	4E-03	M+ACN+H	ESI+
193	2,79	243,07	Piceatannol	Phenylpropanoid	C14H12O4	-3,85	86,39	8E-05	M-H	ESI-
194	2,79	271,10	3 4-Dihydroxystyrene	Phenylpropanoid	C8H8O2	-3,53	92,32	1E-04	2M-H	ESI-
195	3,02	155,03	Shikimate	Amino acid	C7H10O5	-4,98	91,60	3E-04	M-H2O-H	ESI-
196	3,60	665,24	Flavaprenin 7 4-diglucoside	Flavonoid	C32H40O15	-3,56	80,58	5E-03	M+H	ESI+
197	3,66	649,14	Delphinidin 3-O-3 6-O-dimalonylglucoside	Flavonoid	C27H30O17	-2,23	96,55	8E-05	M+H, M+Na, M+K	ESI+
198	3,60	369,12	3-Oxooctanoyl-acp	Lipid	C8H13O2S	-1,72	88,48	2E-04	2M+Na	ESI+
199	5,25	615,35	Astaxanthin diester	Terpenoid	C42H50O6	0,32	80,62	2E-02	M+H-2H2O	ESI+

200	3,47	636,24	N-Acetylneuraminate	Carbohydrate	C11H19NO9	-1,59	86,43	7E-03	2M+NH4	ESI+
201	4,31	603,20	Acanthoside B	Phenylpropanoid	C28H36O13	-3,06	93,56	6E-03	M+Na, M+K	ESI+
202	3,49	389,06	Chlorogenic acid	Phenylpropanoid	C16H18O9	-3,37	85,05	7E-06	M+Cl	ESI-
203	3,91	449,11	2 3 4 4 6-Peptahydroxychalcone 4-O-glucoside	Flavonoid	C21H22O11	-1,77	92,01	4E-08	M-H	ESI-
204	8,28	529,28	Abscisate	Terpenoid	C15H20O4	-4,58	96,57	6E-03	2M+H	ESI+
205	4,34	593,15	Scolymoside	Flavonoid	C27H30O15	-1,12	95,40	8E-07	M-H	ESI-
206	4,32	287,05	2-Hydroxygenistein	Flavonoid	C15H10O6	-1,34	99,38	3E-05	M+H	ESI+
207	3,57	367,10	1-O-Sinapoyl-beta-D-glucose	Phenylpropanoid	C17H22O10	-1,76	96,52	3E-05	M-H2O-H	ESI-
208	0,53	617,17	Thiomethyladenosine	Terpenoid	C11H15N5O3S	-1,65	91,06	5E-07	2M+Na	ESI+
209	3,60	177,05	Ferulic acid	Phenylpropanoid	C10H10O4	0,56	98,61	2E-04	M+H-H2O	ESI+
210	3,35	575,17	cyclo-Dopa-glucuronylglucoside	Amino acid	C21H27NO15	1,57	79,79	1E-04	M+ACN+H	ESI+
211	5,05	530,38	6alpha-Hydroxy-castasterone	Terpenoid	C28H50O5	2,33	90,44	1E-04	M+ACN+Na	ESI+
212	5,78	780,42	Corydaline	Alkaloid	C22H27NO4	4,26	93,88	4E-06	2M+ACN+H	ESI+
213	2,39	516,23	Carnosine	Amino acid	C9H14N4O3	-0,07	91,89	4E-03	2M+ACN+Na	ESI+
214	4,98	412,36	5 7 22 2428-Ergostatetraenol	Terpenoid	C28H42O	-3,98	98,92	5E-04	M+NH4	ESI+
215	2,65	179,03	3-3 4-Dihydroxyphenylpyruvate	Amino acid	C9H8O5	2,34	94,04	3E-06	M+H-H2O	ESI+
216	3,76	648,19	Raloxifene-6-glucuronide	Flavonoid	C34H35NO10S	1,65	79,55	1E-05	M-H	ESI-
217	2,98	186,05	Indolepyruvate	Amino acid	C11H9NO3	-0,48	97,43	2E-04	M+H-H2O	ESI+
218	3,73	391,10	5-O-Feruloylquinic acid	Phenylpropanoid	C17H20O9	-4,09		2E-05	M+H, M+Na, M+K	ESI+
219	4,59	578,19	Dalpaniculin	Flavonoid	C25H28O13	-3,32	95,18	2E-03	M+ACN+H	ESI+
220	5,17	797,28	S-adenosyl-L-methionine	Amino acid	C15H23N6O5S	2,24	92,32	5E-04	2M-H	ESI-
221	4,38	621,18	Pelargonidin 3-6-p-coumaroylglucoside	Flavonoid	C30H27O12	0,79	88,21	3E-02	M+ACN+H	ESI+
222	5,03	567,13	Irigenin 7-O-glucoside	Flavonoid	C24H26O13	-2,04	77,13	1E-02	M+FA-H	ESI-
223	4,22	848,09	acetyl-CoA	Carbohydrate	C23H38N7O17P3S	-1,10	76,59	2E-02	M+K	ESI+
224	4,85	417,25	5R-6-Hydroxy-5-isopropenyl-2-methylhexanoate	Terpenoid	C10H18O3	-3,65	89,04	4E-05	2M+FA-H	ESI-

225	0,72	744,25	Dihydrochelirubine	Alkaloid	C21H17NO5	-0,84	88,09	5E-02	2M+NH4	ESI+
226	3,20	373,13	3-Ketosucrose	Carbohydrate	C12H20O11	-0,80	75,15	2E-03	M+CH3OH+H	ESI+
227	3,87	447,15	Benzyl alcohol beta-D-xylopyranosyl1->6-beta-D-glucopyranoside	Phenylpropanoid	C18H26O10	-3,86	80,22	4E-03	M+FA-H	ESI-
228	2,82	748,09	Cyanidin-3-O-6-O-malonyl-2-O-glucuronylglucoside	Flavonoid	C30H31O20	4,03	85,60	4E-03	M+K-2H	ESI-
229	5,50	379,30	Carboxyspermidine	Amino acid	C8H19N3O2	4,08	98,06	9E-03	2M+H	ESI+
230	3,59	295,08	Dehypoxanthine futalosine	Terpenoid	C14H16O7	-3,73	84,43	5E-02	M-H	ESI-
231	3,13	383,16	2-amino-2 3 7-trideoxy-D-lyxo-hept-6-ulosonic acid	Amino acid	C7H13NO5	-3,61	74,61	2E-03	2M+H	ESI+
232	4,73	603,08	UDP-2-acetamido-3-amino-2 3-dideoxy-alpha-D-glucuronate	Carbohydrate	C17H26N4O17P2	4,60	78,53	1E-02	M+H-H2O	ESI+
233	3,66	340,19	Demissine	Alkaloid	C50H83NO20	4,71	61,56	6E-05	M+3H	ESI+
234	5,09	493,29	Thiobinupharidine	Alkaloid	C30H42N2O2S	-0,40	67,95	1E-02	M-H	ESI+
235	4,90	397,22	Decanoyl-acp	Lipid	C10H19OS	-0,52	90,40	5E-04	2M+Na	ESI+
236	12,19	355,07	Isopentenyl phosphate	Terpenoid	C5H11O4P	0,52	70,98	3E-02	2M+Na	ESI+
237	4,85	433,21	Methyl 9 12 13 15-bisepidioxy-16-hydroperoxy-10-octadecenoate	Lipid	C19H32O8	-3,53	90,10	3E-02	M+FA-H	ESI-
238	12,19	575,10	trans-Zeatin riboside diphosphate	Terpenoid	C15H23N5O11P2	2,94	62,04	2E-02	M+ACN+Na	ESI+
239	6,76	839,20	5 3 5-Trihydroxy-3 6 7 8 4-pentamethoxyflavone	Flavonoid	C20H20O10	-0,09	93,94	4E-04	2M-H	ESI-
240	3,86	328,12	3-Epimacronine	Alkaloid	C18H19NO5	-3,13	94,74	2E-05	M-H	ESI-
241	0,83	308,98	D-ribose 1 5-bisphosphate	Carbohydrate	C5H12O11P2	-3,24	92,16	1E-05	M-H	ESI-
242	14,39	253,22	9Z-Hexadecenoic acid	Lipid	C16H30O2	-4,01	83,50	1E-03	M-H	ESI-
243	2,64	160,08	indol-3-ylacetaldehyde	Amino acid	C10H9NO	-1,87	89,20	2E-02	M+H	ESI+
244	3,44	367,27	Phytoceramide	Lipid	C19H38NO4	-2,05	88,55	1E-02	M+Na	ESI+
245	14,39	277,22	a-Linolenic acid	Lipid	C18H30O2	-3,10	82,44	1E-06	M-H	ESI-
246	1,70	137,05	Hypoxanthine	Nucleotid	C5H4N4O	1,17	93,14	7E-04	M+H	ESI+
247	3,47	490,17	Demethylalangiside	Alkaloid	C24H29NO10	-2,04	88,99	2E-06	M-H	ESI-

248	3,01	457,13	Enzyme N6-(dihydrolipoyl)lysine	Carbohydrate	C8H16NOS2	3,14	87,38	2E-06	2M+FA-H	ESI-
249	3,98	314,10	Hippeastrine	Alkaloid	C17H17NO5	-2,52	82,56	1E-05	M-H	ESI-
250	3,68	316,12	S-Hercyn-2-yl-L-cysteine S-oxide	Amino acid	C12H21N4O5S	-2,55	93,24	9E-06	M+H-H2O, M+K	ESI+
251	4,04	462,18	Morphine-3-glucuronide	Alkaloid	C23H27NO9	-1,31	94,16	3E-05	M+H, M+Na	ESI+
252	4,41	454,04	3-Oxalomalate	Carbohydrate	C6H6O8	-4,06	88,92	1E-04	2M+ACN+H	ESI+
253	4,67	592,12	Cyanidin 3-6-p-caffeoyl glucoside	Flavonoid	C30H27O14+	0,91	71,84	9E-03	M-H2O-H	ESI-
254	4,63	308,15	N-Succinyl-LL-2 6-diaminoheptanedioate	Amino acid	C11H18N2O7	1,84	88,57	1E-04	M+NH4, 2M+H	ESI+
255	2,16	305,16	Feruloylagmatine	Phenylpropanoid	C15H22N4O3	-3,30	86,28	9E-06	M-H	ESI-
256	4,63	597,21	Pelargonidin 3-O-rutinoside	Flavonoid	C27H31O14	3,03	96,50	2E-06	M+NH4	ESI+
257	0,57	423,06	3-2-Carboxyethenyl-cis cis-muconate	Phenylpropanoid	C9H8O6	2,06	91,77	4E-03	2M-H	ESI-
258	3,61	430,09	Cyanidin 5-O-glucoside	Flavonoid	C21H21O11+	3,21	78,04	1E-08	M-H2O-H	ESI-
259	0,51	88,04	L-alanine	Amino acid	C3H7NO2	-4,67	95,94	7E-04	M-H	ESI-
260	3,20	437,20	L-Glutamine	Amino acid	C5H10N2O3	3,85	92,71	4E-06	3M-H	ESI-
261	13,05	399,35	Dodecanoic acid	Lipid	C12H24O2	-2,82	87,92	2E-02	2M-H	ESI-
262	3,92	504,19	Alangiside	Alkaloid	C25H31NO10	-1,99	86,78	1E-05	M-H	ESI-
263	3,91	342,13	Sinapoyltyramine	Amino acid	C19H21NO5	-3,33	83,17	3E-05	M-H	ESI-
264	3,61	367,15	gibberellin A37	Terpenoid	C20H26O5	-1,25	79,45	3E-04	M+Na-2H	ESI-
265	3,47	328,12	Melicopicine	Alkaloid	C18H19NO5	-3,11	85,86	9E-06	M-H	ESI-
266	3,84	474,18	Codeine-6-glucuronide	Alkaloid	C24H29NO9	-1,46	95,55	1E-06	M-H	ESI-
267	3,20	490,17	Demethylisoalangiside	Alkaloid	C24H29NO10	-2,09	91,02	5E-08	M-H	ESI-
268	5,39	532,22	Copal-8-ol diphosphate	Terpenoid	C20H38O8P2	-3,54	91,38	3E-02	M+ACN+Na	ESI+
269	6,64	263,16	1-3 4-Dihydroxyphenyl-5-hydroxy-3-decanone	Phenylpropanoid	C16H24O4	-3,14	94,96	8E-03	M+H-H2O	ESI+
270	9,26	328,20	sarpagine	Alkaloid	C19H22N2O2	0,46	85,89	4E-05	M+NH4	ESI+
271	9,17	233,15	Germacrene A acid	Terpenoid	C15H22O2	-3,53	84,48	3E-03	M-H	ESI-
272	9,47	277,18	6-Gingerol	Phenylpropanoid	C17H26O4	-1,49	89,13	1E-07	M+H-H2O	ESI+

273	2,77	898,62	Thermozeaxanthin	Terpenoid	C54H80O8	-3,38	54,36	3E-04	M+ACN+H	ESI+
274	3,53	558,14	Flavonol 3-O-beta-D-glucosyl-1->2-beta-D-glucoside	Flavonoid	C27H25O13	-1,06	82,02	2E-04	M+H	ESI+
275	3,33	417,16	loganate	Terpenoid	C16H23O10	4,42	96,04	5E-04	M+ACN+H	ESI+
276	6,28	884,07	Oxalyl-CoA	Carbohydrate	C23H36N7O19P3S	3,48	87,41	1E-03	M+2Na-H	ESI+
277	6,81	318,30	Phytosphingosine	Lipid	C18H39NO3	-4,12	99,33	6E-05	M+H-H2O, M+H	ESI+
278	5,07	382,11	indolylmethyl desulfoglucosinolate	Amino acid	C16H20N2O6S	-2,17	79,84	6E-03	2M+3H2O+2H	ESI+
279	9,87	383,26	Decanoate	Lipid	C10H20O2	1,87	94,93	1E-04	2M+K	ESI+
280	6,38	181,12	Anethole	Phenylpropanoid	C10H12O	-1,15	98,38	1E-04	M+CH3OH+H	ESI+
281	9,60	313,27	Linoleate	Lipid	C18H32O2	-4,78	92,76	2E-02	M+CH3OH+H	ESI+
282	6,43	671,39	Lobelanine	Alkaloid	C22H25NO2	4,65	78,05	4E-03	2M+H	ESI+
283	5,88	281,13	Dihydrobiopterin	Folate	C9H13N5O3	-2,81	97,97	6E-04	M+ACN+H	ESI+
284	0,49	406,92	3-Phosphohydroxypyruvate	Carbohydrate	C3H5O7P	-2,27	81,90	4E-03	2M+K	ESI+
285	4,64	775,70	Tetracosanoic acid	Lipid	C24H48O2	4,22	76,47	2E-05	2M+K	ESI+
286	9,51	421,23	ecgonine methyl ester	Alkaloid	C10H17NO3	0,40	94,16	4E-02	2M+Na	ESI+
287	3,55	488,16	7 8-Dihydrofolate	Folate	C19H21N7O6	4,11	87,50	7E-03	M+FA-H	ESI-
288	3,69	468,16	THF	Carbohydrate	C19H23N7O6	3,22	86,15	2E-04	M+Na	ESI+
289	6,51	339,25	aphidicolin	Terpenoid	C20H34O4	-3,23	94,40	2E-03	M+H	ESI+
290	1,27	604,07	GDP-L-gulose	Carbohydrate	C16H25N5O16P2	-1,80	79,29	4E-07	M-H	ESI-
291	4,65	282,11	N-Phenylacetylphenylalanine	Phenylpropanoid	C17H17NO3	-3,04	87,21	3E-05	M-H	ESI-
292	5,03	282,11	Morphinone	Alkaloid	C17H17NO3	-3,01	85,76	3E-05	M-H	ESI-
293	0,89	180,07	L-Tyrosine	Amino Acid	C9H11NO3	-3,82	89,74	4E-02	M-H	ESI-
294	1,26	565,05	UDP-glucose	Carbohydrate	C15H24N2O17P2	-0,93	94,09	7E-08	M-H	ESI-
295	4,63	465,29	Terpendole G	Terpenoid	C28H37NO4	3,04	82,60	2E-04	2M+3H2O+2H	ESI+
296	4,47	812,36	Crinine	Alkaloid	C16H17NO3	4,02	91,86	5E-05	3M-H	ESI-
297	5,32	483,22	Zeatin	Terpenoid	C10H13N5O	0,08	87,88	2E-03	2M+FA-H	ESI-
298	1,33	549,05	UDP-Rhamnose	Carbohydrate	C15H24N2O16P2	-1,78	81,04	2E-05	M-H	ESI-

299	5,42	743,09	Unsaturated digalacturonate	Carbohydrate	C12H16O12	0,88	67,39	6E-05	2M+K	ESI+	
300	1,83	203,14	Pseudoecgonine	Alkaloid	C9H15NO3	-2,89	98,60	5E-04	M+H, M+NH4	ESI+	
301	2,02	249,12	6-hydroxymelatonin	Amino acid	C13H16N2O3	2,72	83,35	4E-05	M+H	ESI+	
302	0,50	393,00	Inosine diphosphate	Nucleotid	C10H14N4O11P2	0,84	66,79	5E-03	M+H-2H2O	ESI+	
303	2,77	295,14	R-mevalonic acid	Terpenoid	C6H12O4	-4,15	93,01	8E-03	2M-H	ESI-	
304	4,19	342,13	Codeinone	Alkaloid	C18H19NO3	-4,07	81,33	3E-02	M+FA-H	ESI-	
305	4,25	504,19	Isoalangiside	Alkaloid	C25H31NO10	-1,84	76,44	3E-03	M-H	ESI-	
306	3,22	788,18	Delphinidin 3-glucoside 5-caffoyl-glucoside	Flavonoid	C36H37O20+	0,70	69,83	2E-03	M-H	ESI-	
307	11,94	599,41	Capsorubin	Terpenoid	C40H56O4	-1,86	67,90	7E-05	M-H	ESI-	
308	5,88	629,26	Sinapyl alcohol	Phenylpropanoid	C11H14O4	-0,03	83,87	4E-06	3M-H	ESI-	
309	6,51	864,18	Monodemalonylsalvianin	Flavonoid	C39H39O21	3,63	79,54	1E-02	M+Na-2H	ESI-	
310	11,35	400,32	13Z 16Z-Docosadienoic acid	Lipid	C22H40O2	3,06	93,16	4E-02	M+ACN+Na	ESI+	
311	4,98	617,26	Hordatine B-like compounds	Amino acid	C29H40N8O5	-0,45	93,75	4E-12	M+K-2H	ESI-	
312	11,63	423,29	Volicitin	Lipid	C23H38N2O5	0,98	90,42	1E-02	M+H	ESI+	
313	5,43	632,04	GDP-4-dehydro-6-deoxy-D-mannose	Carbohydrate	C16H23N5O15P2	-0,63	86,29	4E-04	M+2Na-H	ESI+	
314	6,70	675,41	ent-7alpha-Hydroxykaur-16-en-19-oic acid	Terpenoid	C20H30O3	4,52	95,26	4E-02	2M+K	ESI+	
315	3,31	359,13	Hippurate	Amino acid	C9H9NO3	4,89	81,86	4E-05	2M+H	ESI+	
316	4,41	577,16	Naringenin 5 7-di-O-glucoside	Flavonoid	C27H32O15	-1,93	86,98	4E-03	M-H2O-H	ESI-	
317	0,87	391,03	N-5-phosphonato-ß-D-ribosylanthranilate	Amino acid	C12H13NO9P	1,96	91,31	5E-02	M+FA-H	ESI-	
318	5,35	330,13	Longifolonine	Alkaloid	C17H15NO4	-4,96	96,26	4E-03	M+CH3OH+H	ESI+	
319	4,61	568,22	Aclacinomycin T	Terpenoid	C30H35NO10	-2,44	74,99	5E-02	M-H	ESI-	
320	4,17	601,28	Atractyloside G 2-O-i²-D-glucopyranoside	Terpenoid	C27H46O13	-4,75	82,29	1E-05	M+Na	ESI+	
321	3,28	326,10	Terrestriamide	Phenylpropanoid	C18H17NO5	-3,28	84,77	9E-05	M-H	ESI-	
322	2,75	443,19	Dihydrophaseic acid 4-O-ß-D-glucoside	Terpenoid	C21H32O10	-2,39	85,02	6E-03	M-H	ESI-	
323	6,54	591,35	Cucurbitacin B	Terpenoid	C32H46O8	-3,95	83,11	3E-02	M+CH3OH+H	ESI+	
324	4,16	312,12	N-feruloyltyramine	Phenylpropanoid	C18H19NO4	-3,27	81,14	2E-03	M-H	ESI-	
325	5,42	713,08	2 3-Cyclic GMP	Nucleotid	C10H12N5O7P	-1,66	96,72	3E-04	2M+Na	ESI+	

326	11,77	376,32	Arachidic acid	Lipid	C20H40O2	-1,98	96,33	1E-02	M+ACN+Na	ESI+
327	3,96	724,19	Cyanidin 5-O-beta-D-glucoside 3-O-beta-D-sambubioside	Flavonoid	C32H39O20	0,93	87,78	3E-02	M-H2O-H	ESI+
328	6,21	617,22	Bisdemethoxycurcumin	Phenylpropanoid	C19H16O4	2,82	98,36	3E-04	2M+H	ESI+
329	8,87	369,24	Taxa-420 1112-dien-5alpha-acetoxy-10beta-ol	Terpenoid	C22H34O3	0,18	95,55	4E-02	M+Na	ESI+
330	9,97	194,12	Tryptophol	Amino acid	C10H11NO	-3,97	93,43	5E-03	M+CH3OH+H	ESI+
331	9,11	347,22	swainsonine	Alkaloid	C8H15NO3	0,86	94,26	6E-05	2M+H	ESI+
332	0,55	247,14	D-Octopine	Amino acid	C9H18N4O4	-3,97	96,52	1E-06	M+H	ESI+
333	3,08	336,19	Octadecenoyl-acp	Lipid	C18H33OS	4,79	93,75	2E-09	M+K	ESI+
334	3,16	278,07	5 6 7 8-Tetrahydrobiopterin	Folate	C9H15N5O3	-0,56	88,41	0E+00	M+K-2H	ESI-
335	7,95	215,16	Butyraldehyde	Carbohydrate	C4H8O	-1,55	96,47	1E-14	3M-H	ESI-
336	2,49	307,09	S-Succinyldihydrolipoamide-E	Carbohydrate	C12H20NO4S2	-0,69	78,01	0E+00	M+H	ESI+
337	4,49	600,33	all-trans-hexaprenyl diphosphate	Folate	C30H52O7P2	3,23	89,84	2E-10	2M+3H2O+2H	ESI+
338	0,55	539,14	Cellodextrin	Carbohydrate	C18H32O16	-2,36	88,75	4E-07	M+Cl	ESI-
339	0,54	867,24	Pelargonidin 3-glucoside	Flavonoid	C21H21O10	1,14	89,94	7E-12	2M+H	ESI+
340	1,08	153,04	Xanthine	Alkaloid	C5H4N4O2	-3,06	85,12	6E-12	M+H	ESI+
341	2,49	294,06	Dihydroneopterin	Folate	C9H13N5O4	-0,13	97,49	1E-10	M+K	ESI+
342	8,89	294,24	Stearidonic acid	Lipid	C18H28O2	-3,27	74,30	7E-03	M+NH4	ESI+
343	1,91	253,13	8-propyloxycaffeine	Phenylpropanoid	C11H16N4O3	-1,63	67,85	4E-05	M+H	ESI+
344	8,95	403,33	4 4-Diaponeurosporene	Terpenoid	C30H42	-3,31	84,72	1E-03	M+H	ESI+
345	9,06	495,34	Capsidiol	Terpenoid	C15H24O2	-2,60	84,46	7E-04	2M+Na	ESI+
346	12,81	140,07	L-Valine	Amino acid	C5H11NO2	0,28	93,87	2E-02	M+Na	ESI+
347	5,27	697,07	3 5-Cyclic AMP	Nucleotid	C10H12N5O6P	-4,21	90,21	7E-06	2M+K	ESI+
348	5,38	528,04	UDP-L-Ara4FN	Carbohydrate	C15H23N3O16P2	1,29	86,38	2E-03	M+H-2H2O	ESI+
349	4,37	187,10	Z-3-Hexen-1-ol acetate	Lipid	C8H14O2	-4,78	91,20	1E-08	M+FA-H	ESI-
350	4,86	577,15	Flavonol 3-O-D-xylosylglucoside	Flavonoid	C26H28O12	-2,52	73,20	1E-04	M+FA-H	ESI-
351	13,17	465,37	24-Hydroxy-beta-amyrin	Terpenoid	C30H50O2	-3,65	91,52	7E-03	M+Na	ESI+

352	6,12	639,20	Salidroside	Amino acid	C14H20O7	-2,44	87,67	6E-03	2M+K	ESI+
353	6,89	663,24	PR-toxin	Terpenoid	C17H20O6	-1,49	81,91	2E-03	2M+Na	ESI+
354	2,83	429,18	Indole-3-butyric acid	Hormone	C12H13NO2	-0,91	96,68	2E-03	2M+Na	ESI+
355	5,42	833,41	vincaleukoblastine	Alkaloid	C46H58N4O9	3,55	83,50	2E-03	M+Na	ESI+
356	6,19	613,27	Galanthamine	Alkaloid	C17H21NO3	-1,28	94,31	4E-06	2M+K	ESI+
357	3,44	173,01	Citrate	Carbohydrate	C6H8O7	-3,71	92,38	1E-02	M-H2O-H	ESI-
358	3,97	123,08	Phenylethyl alcohol	Amino acid	C8H10O	-0,83	91,45	4E-02	M+H	ESI+
359	5,23	747,34	4 21-dehydrogeissoschizine	Alkaloid	C21H23N2O3	2,27	85,47	4E-04	2M+FA-H	ESI-
360	4,86	516,12	2-Hydroxypropyl-ThPP	Carbohydrate	C15H25N4O8P2S	1,03	88,18	4E-03	M+CH3OH+H	ESI+
361	5,13	747,34	4 21-Dehydrocorynantheine aldehyde	Alkaloid	C21H23N2O3	3,38	94,08	5E-05	2M+FA-H	ESI-
362	11,17	525,37	Sphingomyelin	Lipid	C24H49N2O6P	2,04	59,70	7E-03	M+CH3OH+H	ESI+
363	4,98	279,11	Indole	Amino acid	C8H7N	-4,78	91,35	6E-06	2M+FA-H	ESI-
364	4,97	297,12	Phosphoguanidinoacetate	Amino acid	C10H18N4O5	2,34	91,31	5E-08	M+H-2H2O, M+Na	ESI+
365	4,98	677,40	Gibberellin A12 aldehyde	Terpenoid	C20H28O3	-1,87	79,81	4E-07	2M+FA-H	ESI-
366	5,18	649,29	N1 N5 N10-Tricaffeoyl spermidine	Phenylpropanoid	C34H37N3O9	-1,01	92,64	2E-09	M+NH4	ESI+
367	0,51	161,06	1-3 4-Dihydroxyphenyl-1-decene-3 5-dione	Phenylpropanoid	C16H20O4	-4,19	90,87	1E-04	M+2Na	ESI+
368	4,97	293,13	2-Phenylacetamide	Amino acid	C8H9NO	-2,56	98,51	5E-03	2M+Na	ESI+
369	3,04	490,28	trans-Dodec-2-enoyl-acp	Lipid	C12H21OS	3,61	90,75	2E-02	2M+ACN+Na	ESI+
370	4,47	794,34	Aclacinomycin N	Terpenoid	C42H55NO15	-1,59	75,94	3E-03	M-H2O-H	ESI-
371	3,08	300,11	trans-Hex-2-enoyl-acp	Lipid	C6H9OS	4,57	55,05	3E-03	2M+ACN+H	ESI+
372	4,19	287,05	Leucodelphinidin	Flavonoid	C15H14O8	-2,11	98,81	2E-02	M+H-2H2O, M+NH4	ESI+
373	4,46	415,34	Phylloquinone	Terpenoid	C31H46O2	4,69	81,42	2E-06	M+H-2H2O	ESI+

S2 Table. Metabolites of secondary metabolism pathways represented by the heatmap (Fig 2). Each metabolite is described by ionization mode, identification ("Level 2"). Different letters indicate significant differences based Tuckey test (p-value <0.05).

		Water	CCF	BABA	*Ulva* extract
**Phenylpropanoid compounds**					
ESI+	Anethole	b	a	a	a
ESI-	Feruloylagmatine	b	a	a	b
ESI+	6-Gingerol	b	a	a	b
ESI+	1,3,4-Dihydroxyphenyl-5-hydroxy-3-decanone	b	a	ab	ab
ESI-	N-feruloyltyramine	ab	a	ab	b
ESI-	Sinapyl alcohol	ab	a	bc	c
ESI+	N1-N5-N10-Tricaffeoyl spermidine	a	a	b	c
ESI+	1,3,4-Dihydroxyphenyl-1-decene-3,5-dione	a	a	b	b
ESI+	Bisdemethoxycurcumin	a	a	a	b
ESI-	3,2-Carboxyethenyl-cis-cis-muconate	ab	a	a	b
ESI-	Terrestriamide	bc	a	ab	c
ESI+	Sinapaldehyde	a	a	b	a
ESI-	4-O-beta-D-Glucosyl sinapate	a	ab	b	a
ESI-	Caffeyl alcohol	a	b	b	a
ESI+	Curcumin	a	ab	b	a
ESI+	Coniferyl alcohol	b	b	b	a
ESI-	L-Quinate	b	b	b	a
ESI-	p-Coumaroyl quinic acid	b	c	c	a
ESI+	Scopolin	b	c	c	a
ESI-	4-p-Coumaroylquinic acid	b	c	c	a
ESI-	Caffeoyl quinic acid	b	c	c	a
ESI-	1,3-Dicaffeoylquinic acid	b	c	c	a
ESI+	N-caffeoylputrescine	a	b	b	ab
ESI+	p-Coumaraldehyde	a	bc	c	ab
ESI-	Shikimate	a	b	b	a
ESI-	5-O-Caffeoylshikimic acid	a	b	b	a
ESI+	Scopoletin	a	b	b	a
ESI-	5-Hydroxyferulic acid methyl ester	a	b	b	a
ESI+	Coniferyl acetate	a	b	b	a
ESI-	Caffeic acid	a	b	b	a
ESI-	3,4-Dihydroxystyrene	a	b	b	a
ESI-	Piceatannol	a	b	b	a
ESI+	5-O-Feruloylquinic acid	ab	b	b	a
ESI-	Chlorogenic acid	a	b	b	a
ESI+	Acanthoside B	ab	b	b	a
ESI-	1-O-Sinapoyl-beta-D-glucose	a	b	b	a

ESI+	Ferulic acid	ab	bc	c	a
ESI+	Methyleugenol	c	bc	ab	a
ESI-	N-Phenylacetylphenylalanine	ab	b	a	ab
ESI+	Syringin	a	b	a	a
ESI-	Caffeic acid-3-glucoside	a	b	ab	ab
ESI+	4-Coumaroyl-3-hydroxyagmatine	a	b	ab	ab
**Flavonoid compounds**					
ESI-	Kaempferitrin	a	b	b	a
ESI+	Quercetin 3-O-(6-O-malonyl-beta-D-glucoside)	a	b	b	a
ESI+	Delphinidin 3-O-(6''-O-malonyl)-beta-D-glucoside	a	b	b	ab
ESI-	Quercetin 3-(2G-xylosylrutinoside)	a	b	b	a
ESI-	Baimaside	a	b	b	a
ESI-	Kaempferol 3-sophorotrioside	a	b	b	a
ESI+	Delphinidin 3-O-3'',6''-O-dimalonylglucoside	ab	bc	c	a
ESI-	Dihydroquercetin	a	b	b	a
ESI-	Vitexin 2''-O-beta-L-rhamnoside	a	b	b	a
ESI+	3-O-Methylquercetin	a	b	b	a
ESI-	Rutin	a	b	b	a
ESI+	Dihydromyricetin	ab	bc	c	a
ESI-	Prunin	b	c	c	a
ESI-	Ayarin	b	c	c	a
ESI+	3,7-Di-O-methylquercetin	a	b	b	a
ESI-	2',3,4,4',6'-Peptahydroxychalcone 4'-O-glucoside	a	b	b	a
ESI-	Scolymoside	a	b	b	a
ESI-	Rhoifolin	b	b	b	a
ESI+	Hesperetin 7-O-glucoside	a	a	a	a
ESI+	Quercetin 3-sulfate	a	b	ab	ab
ESI+	Myricetin	ab	b	a	ab
ESI+	Xanthohumol	b	b	a	a
ESI+	Delphinidin 3-O-(6-caffeoyl-beta-D-glucoside)	ab	b	ab	a
ESI+	Leucodelphinidin	ab	a	b	ab
ESI-	Cyanidin-3-O-(6''-O-malonyl-2''-O-glucuronyl)glucoside	a	a	b	ab
ESI+	Pelargonidin 3-(6-p-coumaroyl)glucoside	a	ab	b	ab
ESI-	Flavonol 3-O-D-xylosylglucoside	b	a	b	b
ESI+	Pelargonidin 3-glucoside	b	a	b	b
ESI-	Monodemalonylsalvianin	ab	a	ab	b
ESI-	Delphinidin 3-glucoside 5-caffoyl-glucoside	ab	a	ab	b
ESI-	Cyanidin 5-O-beta-D-glucoside 3-O-beta-D-sambubioside	a	a	a	a
ESI-	Cyanidin 3-(6-p-caffeoyl) glucoside	b	a	ab	b
ESI-	Cyanidin 5-O-glucoside	b	a	a	b
ESI+	Pelargonidin 3-O-rutinoside	b	ab	a	b
**Alkaloid compounds**					

ESI+	Galanthamine	a	a	b	b
ESI+	Swainsonine	b	a	b	b
ESI+	Xanthine	b	a	b	b
ESI-	Isoalangiside	ab	a	ab	b
ESI-	Codeinone	a	a	a	a
ESI-	3-Epimacronine	b	a	a	b
ESI-	Alangiside	bc	a	ab	c
ESI-	Demethylalangiside	b	a	a	b
ESI-	Hippeastrine	b	a	a	b
ESI-	Melicopicine	b	a	a	b
ESI-	Demethylisoalangiside	b	a	a	b
ESI-	Codeine-6-glucuronide	b	a	a	b
ESI+	Lobelanine	b	ab	a	ab
ESI+	Ecgonine methyl ester	a	a	a	a
ESI-	Crinine	b	ab	a	b
ESI+	Morphine-3-glucuronide	b	a	a	b
ESI+	Pseudoecgonine	b	b	a	b
ESI-	Chelirubine	a	b	b	a
ESI+	N-Methylanthranilate	a	b	b	a
ESI-	Thiobinupharidine	a	b	b	b
ESI+	Dihydrochelirubine	a	a	a	a
ESI+	L-Hyoscyamine	a	a	a	a
ESI+	Tropate	a	b	b	a
ESI+	Corydaline	a	b	b	a
ESI+	Solasodine	ab	b	b	a
ESI+	Solamargine	ab	b	b	a
ESI+	Solanidine	b	b	b	a
ESI-	Caffeine	b	c	c	a
ESI+	Autumnaline	ab	b	ab	a
ESI+	Demissine	a	a	b	a
ESI+	N-Glucosylnicotinate	ab	bc	c	a
ESI-	Atractyloside D	a	b	b	a
ESI+	13-hydroxylupanine	a	ab	b	a
ESI+	Peimine	a	ab	b	a
ESI-	Morphinone	ab	b	a	ab
ESI+	Ruscopine	a	b	a	a
ESI+	(S)-6-O-Methylnorlaudanosoline	ab	b	a	a
ESI+	Tubulosine	a	b	a	a
ESI+	(S)-N-Methylcanadine	a	b	a	a
ESI+	Berberine	b	ab	ab	a
ESI+	4'-O-Methylnorbelladine	a	b	a	a
ESI+	Terminaline	a	a	a	a
ESI+	Allocryptopine	b	b	b	a

ESI-	3,4-Dihydroxybenzaldehyde	b	b	b	a
ESI+	Deacetoxyvindoline	b	b	b	a
ESI+	(6S)-6-hydroxyhyoscyamine	b	b	b	a
**Terpenoid compounds**					
ESI-	Gibberellin A12 aldehyde	a	a	b	c
ESI-	Capsorubin	a	a	a	b
ESI+	24-Hydroxy-beta-amyrin	b	a	b	b
ESI+	Capsidiol	b	a	b	b
ESI+	4,4'-Diaponeurosporene	b	a	b	b
ESI+	Ent-7alpha-Hydroxykaur-16-en-19-oic acid	a	a	a	a
ESI-	Dihydrophaseic acid 4-O-β-D-glucoside	b	a	b	b
ESI+	loganate	bc	a	ab	c
ESI-	Gibberellin A37	bc	a	ab	c
ESI+	Copal-8-ol diphosphate	c	a	ab	bc
ESI+	Thermozeaxanthin	b	a	ab	b
ESI-	Germacrene A acid	b	a	ab	b
ESI+	Taxa-4(20),11(12)-dien-5alpha-acetoxy-10beta-ol	a	a	a	a
ESI+	Secologanate	a	a	a	a
ESI+	Phylloquinone	a	a	b	ab
ESI-	(5R)-6-Hydroxy-5-isopropenyl-2-methylhexanoate	a	bc	c	ab
ESI-	Dehypoxanthine futalosine	a	ab	b	ab
ESI+	1'-Hydroxy-gamma-carotene glucoside ester	bc	c	b	a
ESI+	10beta,14beta-Dihydroxytaxa-4(20),11-dien-5alpha-yl acetate	b	b	b	a
ESI-	2-C-methyl-D-erythritol 2,4-cyclic diphosphate	c	bc	b	a
ESI+	26-Hydroxybrassinolide	b	b	b	a
ESI+	Yamogenin 3-O-neohesperidoside	b	b	b	a
ESI+	Phytoene	b	b	b	a
ESI+	Torulene	b	b	b	a
ESI+	Costunolide	ab	b	b	a
ESI-	Gentiopicrin	b	c	c	a
ESI+	5,7,22,24(28)-Ergostatetraenol	ab	b	b	a
ESI+	Glycosyl-4,4'-diaponeurosporenoate	ab	b	ab	a
ESI-	Gibberellin A4	b	c	c	a
ESI+	9-cis-10'-Apo-beta-carotenal	a	b	b	a
ESI+	Abscisate	ab	b	b	a
ESI+	Thiomethyladenosine	a	b	b	a
ESI+	Calcitetrol	a	ab	b	a
ESI+	Astaxanthin diester	ab	ab	b	a
ESI+	Xanthoxin	a	b	b	a
ESI-	Dehydrodolichol diphosphate	a	c	bc	ab
ESI+	Secalciferol	a	b	b	a
ESI-	Bruceoside A	a	b	b	a
ESI-	Asperuloside	a	a	a	a

ESI-	Abscisic aldehyde	a	b	b	a
ESI-	Futalosine	a	bc	c	ab
ESI-	Aconitine	a	b	b	ab
ESI+	Plaunotol	a	a	a	a
ESI+	Aphidicolin	b	b	a	ab
ESI-	L-Tyrosine	a	a	a	a
ESI-	(R)-mevalonic acid	ab	ab	a	b
ESI-	Zeatin	b	ab	a	b
ESI+	Dihydrozeatin-O-glucoside	a	b	a	ab
ESI+	6beta,7beta-Dihydroxykaurenoic acid	a	b	a	a
ESI+	Phoenicoxanthin	a	b	a	a
ESI+	Staphyloxanthin	a	b	a	a
ESI+	Isorenieratene	a	b	a	a
ESI+	Presqualene diphosphate	ab	c	a	b
ESI+	Phaseic acid	a	b	ab	ab
ESI+	Trans-Zeatin riboside diphosphate	a	b	ab	b
ESI+	Isopentenyl phosphate	a	b	ab	b

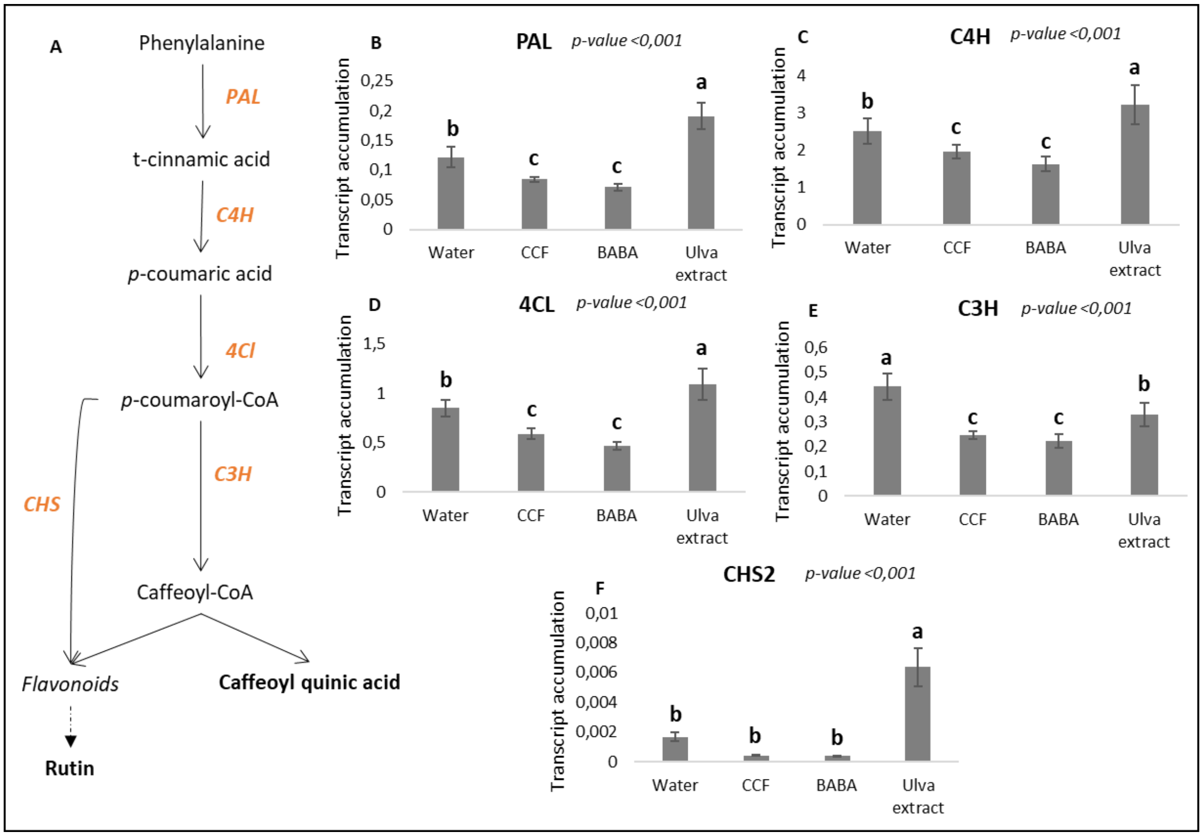

S3 Fig. Phenylpropanoid and derivate pathways and the main genes involved on the biosynthesis of the metabolites. (A).Schematic representation of phenylpropanoid and flavonoid pathways were based on KEGG maps. The target genes (orange bold) regulated by the elicitors were analysed by RT-qPCR. Rutin, caffeoyl quinic acid and α-chaconine (black bold metabolites) were quantified by UPLC-qTOF-MS^{e}.. Solid arrows indicate a direct link between the metabolites and dotted arrows indicate indirect link. **(B-F)** The target genes regulated by the elicitors were analysed by RT-qPCR. The transcript accumulation are illustrated by histograms. Bars represent standard error of two independent trials. The dataset was analysed by linear mixed-effects model fit by REML. Different letters indicate significant differences based on same test (p-value <0.05).

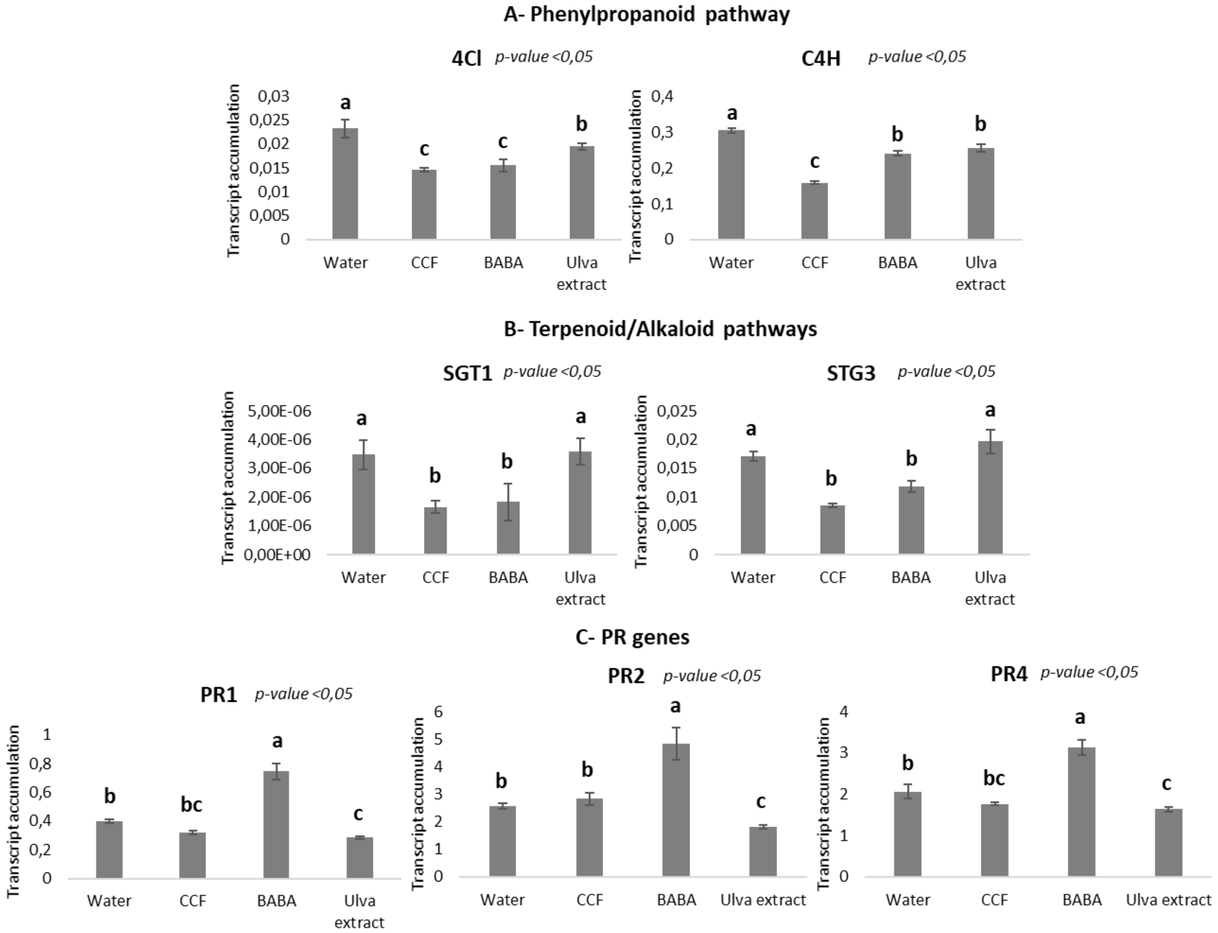

S4 Fig. Complementary transcript analysis of the third trial. Transcript from **(A)** phenylpropanoid pathway **(B)** terpenoid and alkaloid pathways and **(C)** *PR* genes. The target genes regulated by the elicitors were analysed by RT-qPCR. The transcript accumulation are illustrated by histograms. Bars represent standard error of the third independent trial. The dataset was analysed by Linear mixed-effects model fit by REML. Different letters indicate significant differences based on same test (p-value <0.05).

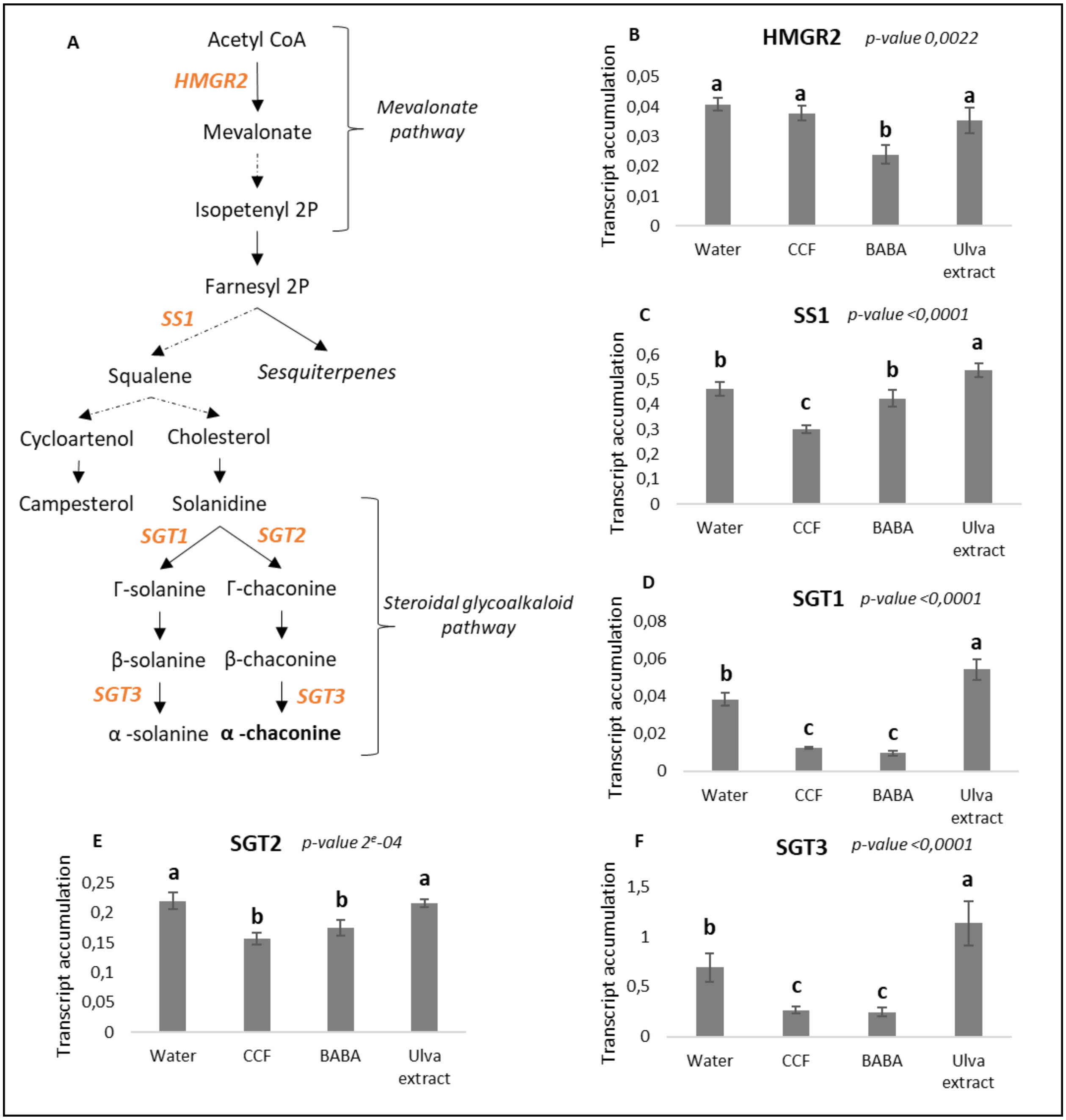

S5 Fig. Alkaloid pathway with the origin of terpenes' biosynthesis (mevalonate pathway) and the main genes involved on the biosynthesis of the metabolites. (A) Schematic representation of terpenoid and alkaloid biosynthesis pathways with focus on steroidal glycoalkaloid pathway were based on KEGG maps. The target genes (orange bold) regulated by the elicitors were analysed by RT-qPCR. α-chaconine (black bold metabolites) were quantified by UPLC-qTOF-MS^{e}. Solid arrows indicate a direct link between the metabolites and dotted arrows indicate indirect link. **(B-F)** The target genes regulated by the elicitors were analysed by RT-qPCR. The transcript accumulation are illustrated by histograms. Bars represent standard error of two independent trials. The dataset was analysed by linear mixed-effects model fit by REML. Different letters indicate significant differences based on same test (p-value <0.05).

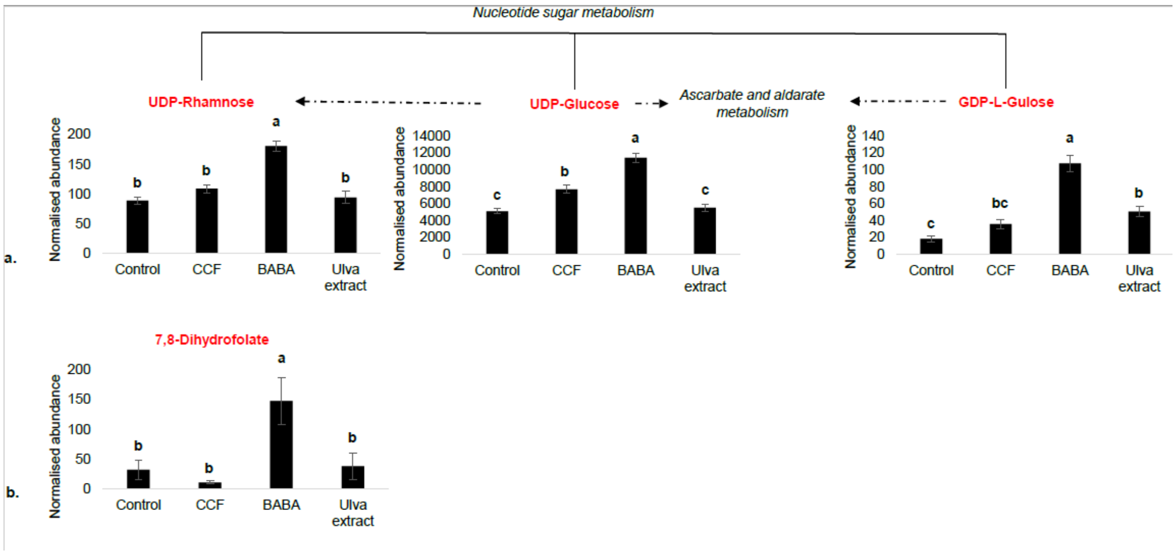

S6 Fig. Metabolites from primary metabolism up-regulated by BABA. The specific up regulations (red bold) are illustrated by histograms of the normalized abundance of metabolites. Different letters indicate significant differences based on Tukey HSD test (p-value<0.05). Bars represent standard error. **(A)** Schematic representation of nucleotide sugar metabolism pathway based on KEGG maps. Dotted arrows indicate indirect link. For **(B)**: 7,8-Dihydrofolate, a folate pathway.

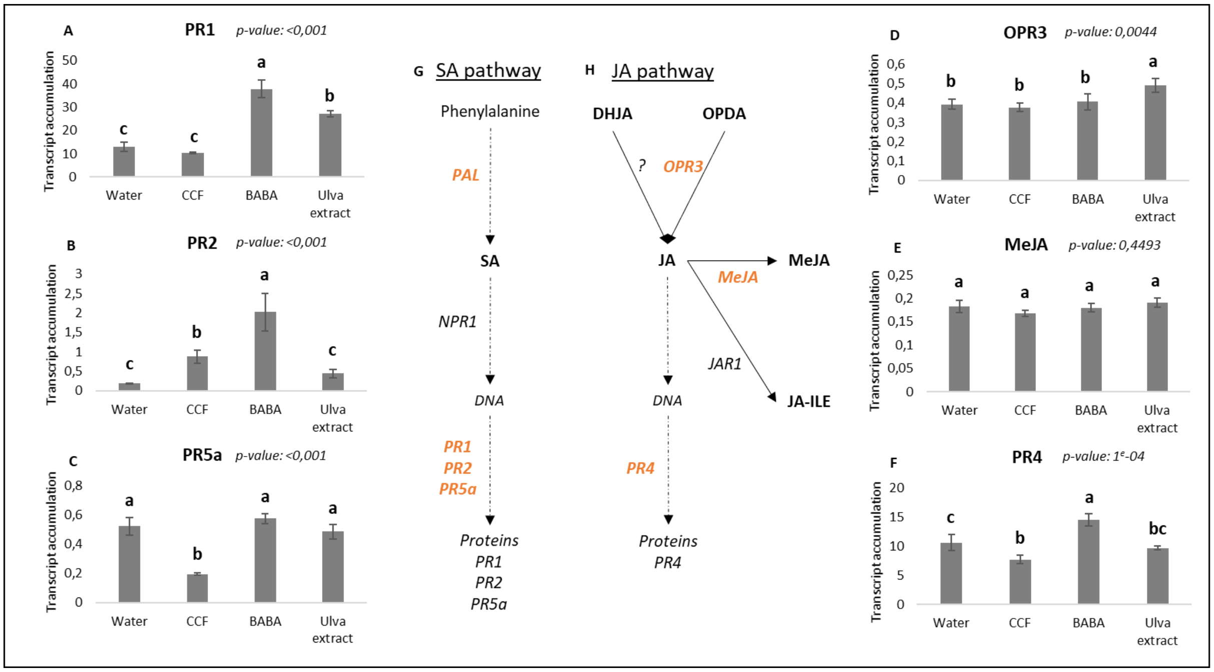

S7 Fig. Phytohormones pathways and PR proteins; the main genes involved on their biosynthesis. The target genes regulated by the elicitors were analysed by RT-qPCR. The transcript accumulation are illustrated by histograms. **(A)** *PR1*, **(B)** *PR2*, **(C)** *PR3*, **(D)** *OPR3*, **(E)** *MeJA* and **(F)** *PR4*. Bars represent standard error of two independent trials. The dataset was analysed by linear mixed-effects model fit by REML. Different letters indicate significant differences based on same test (p-value <0.05). Schematic representations of **(G)** SA and **(H)** JA pathways based on KEGG maps and Ali *et al.* (2018). The target genes (orange bold) regulated by the elicitors were analysed by RT-qPCR. The phytohormones, SA, DHJA, OPDA, JA, MeJA and JA-ILE (black bold metabolites) were quantified by UHPLC-MS/MS. Solid arrows indicate a direct link between the metabolites and dotted arrows indicate indirect link.

Ali, S., Ganai, B. A., Kamili, A. N., Bhat, A. A., Mir, Z. A., Bhat, J. A., et al. (2018). Pathogenesis-related proteins and peptides as promising tools for engineering plants with multiple stress tolerance. *Microbiol. Res.* 212–213, 29–37. doi:10.1016/j.micres.2018.04.008.
